# Non-invasive monitoring of diffuse large B-cell lymphoma by cell-free DNA high-throughput targeted sequencing: analysis of a prospective cohort

**DOI:** 10.1038/s41408-018-0111-6

**Published:** 2018-08-01

**Authors:** Elodie Bohers, Pierre-Julien Viailly, Stéphanie Becker, Vinciane Marchand, Philippe Ruminy, Catherine Maingonnat, Philippe Bertrand, Pascaline Etancelin, Jean-Michel Picquenot, Vincent Camus, Anne-Lise Menard, Emilie Lemasle, Nathalie Contentin, Stéphane Leprêtre, Pascal Lenain, Aspasia Stamatoullas, Hélène Lanic, Julie Libraire, Sandrine Vaudaux, Louis-Ferdinand Pepin, Pierre Vera, Hervé Tilly, Fabrice Jardin

**Affiliations:** 1 INSERM U1245, Centre Henri Becquerel, University of Rouen, Rouen, France; 2 Department of Nuclear Medicine, Centre Henri Becquerel, University of Rouen, Rouen, France; 3 QuantIF–LITIS (EA 4108-FR CNRS 3638), Faculty of Medicine, University of Rouen, Rouen, France; 4 Department of Oncology Genetics, Centre Henri Becquerel, Rouen, France; 5 Department of Pathology, Centre Henri Becquerel, Rouen, France; 6 Department of Clinical Haematology, Centre Henri Becquerel, Rouen, France; 7 Clinical Research Unit, Centre Henri Becquerel, Rouen, France

## Abstract

From a liquid biopsy, cell-free DNA (cfDNA) can provide information regarding basal tumoral genetic patterns and changes upon treatment. In a prospective cohort of 30 diffuse large B-cell lymphomas (DLBCL), we determined the clinical relevance of cfDNA using targeted next-generation sequencing and its correlation with PET scan imaging at the time of diagnosis and during treatment. Using a dedicated DLBCL panel, mutations were identified at baseline for 19 cfDNAs and profiles were consistent with expected DLBCL patterns. Tumor burden-related clinical and PET scan features (LDH, IPI, and metabolic tumor volume) were significantly correlated with the quantity of tumoral cfDNA. Among the four patients presenting additional mutations in their cfDNAs, three had high metabolic tumor volumes, suggesting that cfDNA more accurately reflects tumor heterogeneity than tissues biopsy itself. Mid-treatment, four patients still had basal mutations in their cfDNAs, including three in partial response according to their Deauville scores. Our study highlights the major interests in liquid biopsy, in particular in the context of bulky tumors where cfDNA allows capturing the entire tumoral mutation profile. Therefore, cfDNA analysis in DLBCL represents a complementary approach to PET scan imaging.

## Introduction

The concept of liquid biopsy has been well known for several years mainly among solid tumors, for which it has been possible to demonstrate the presence of circulating tumor cells, circulating tumor DNA (ctDNA) contained in plasma cell-free circulating DNA (cfDNA), and circulating micro-RNAs^1–4^. In healthy subjects, cfDNA arises from the apoptosis of nucleated cells and is found at low levels in the blood, demonstrating that detection of ctDNA requires highly sensitive technologies, such as those currently available with next-generation sequencing (NGS) approaches or digital PCR. In cancer patients, it is known that plasma ctDNA is mainly released by apoptotic tumor cells, but may also be released by necrotic tumor cells or actively secreted by tumor cells^5^, as illustrated by the large range of DNA fragment sizes that are detected (from 150 base pairs to several kilobases).

The value of liquid biopsies was recently highlighted in a few series of diffuse large B-cell lymphoma (DLBCL) patients but also in primary central nervous system lymphoma (PCNSL) cases in whom high-throughput sequencing of a panel of target genes was performed, demonstrating the successful detection of somatic variants in both the tumors and the plasma with similar mutational profiles^6–8^.

Combined immunochemotherapy and targeted therapies have considerably changed the management of lymphomas over the past decade. Nevertheless, response to treatment is often heterogeneous, and while some patients will remain relapse-free after therapy, others experience early disease progression and may develop chemo-refractoriness due to the acquisition of tumor resistance mechanisms and the clonal evolution of tumor cells. Therefore, ctDNA analyses theoretically offer information regarding potentially actionable mutations, clonal evolution, treatment response, and genetic mechanisms of resistance.

Similar to ctDNA detection, ^18^F-fluoro-2-dexoxy-D-glucose (FDG) positron-emission tomography/computed tomography (PET/CT) is a sensitive, non-invasive tool for DLBCL staging, and has been shown to predict therapeutic response and outcomes in this setting. Interim PET (iPET) performed after two cycles of treatment or at mid-treatment has been considered a strong predictor of outcomes using response criteria, such as the Deauville 5-point scale (5-PS) or the reduction in maximum SUV (ΔSUVmax) method^9^. CtDNA detection and PET/CT imaging have been simultaneously reported in two studies using immunoglobulin high-throughput sequencing. These works demonstrated that detection of molecular disease in the plasma may precede PET/CT detection of relapse and improve specificity with similar sensitivity compared with PET/CT^10–12^.

On behalf the LYSA (Lymphoma Study Association), we recently designed and validated a set of 34 genes (termed “Lymphopanel”) selected according to the literature and a whole-exome sequencing study of relapsed/refractory DLBCL patients. The Lymphopanel was informative for 96% of 215 patients enrolled in LYSA trials, highlighting the molecular heterogeneity of subtypes and identifying somatic mutations with therapeutic and prognostic impacts^13^. Its relevance and accuracy for ctDNA was not evaluated.

The primary aim of the current study was to evaluate our Lymphopanel as a liquid biopsy tool in a prospective cohort of DLBCL. The secondary purposes were to determine its modification upon treatment and to correlate baseline and dynamic ctDNA characteristics with PET scan imaging metrics obtained at baseline and during follow-up.

## Subjects and methods

### Study design and patients

Thirty DLBCL or primary mediastinal B-cell lymphoma (PMBL) patients were enrolled prospectively. Biological material at the time of diagnosis, including DNA and RNA from biopsies, blood and bone marrow, was collected before any treatment, as well as clinical features (bone marrow biopsy results, stage according to the Ann Arbor classification, and international prognostic index (IPI) calculation) were recorded. Patients were followed after rituximab-cyclophosphamide-doxorubicin-vincristine-prednisone (R-CHOP) or R-CHOP-like chemotherapies, and blood samples (for cfDNA extraction) were collected at mid-treatment, end of treatment, and 1 year after end of treatment. An ^18^FDG-PET-TDM was performed at the time of diagnosis and during the follow-up (mid-treatment and end of treatment, according to treatment strategies).

Patients provided written informed consent in accordance with the Declaration of Helsinki, and the institutional review board approved the protocol (Registration clinical.gov number: NCT02339805).

### Sample collection and processing

Tumor genomic DNA (gDNA) was isolated from fresh or formalin-fixed paraffin-embedded (FFPE) diagnostic tissue biopsies. Bone marrow samples were collected at the time of diagnosis. From fresh tissue and bone marrow, gDNA was extracted using proteinase K followed by salt and ethanol precipitation and was stored at −20 °C in 10 mM Tris-Cl and 1 mM EDTA (pH 8) buffer. From FFPE tissue, gDNA was extracted using the QIAamp DNA FFPE Tissue Kit (Qiagen, Courtaboeuf, France), according to the manufacturer’s instructions.

CtDNA monitoring was completed on serial plasma samples. For each patient, plasma samples were collected at baseline and interim monitoring was performed on samples obtained at mid-treatment, at the end of treatment, 6 months after the end of treatment, and in case of relapse (Supplementary Fig. S1). Blood was collected in EDTA tubes that were centrifuged for 10 min at 3000 rpm within 3 h of collection. Plasma was aliquoted into 1 mL in microtubes and stored at −80 °C until extraction. After thawing, plasma aliquots (from 1 to 3 mL) were centrifuged for 5 min at 13,000 rpm. Then, cfDNA was extracted from the supernatant using the QIAamp circulating nucleic acid kit (Qiagen) and quantified using QuBit High Sensitivity dsDNA (Thermo Fisher Scientific, Illkirch, France).

### GCB/ABC phenotyping

For tumoral samples, cell of origin (COO) molecular classification was obtained using the Hans algorithm^14^. Immunohistochemistry staining was performed for CD10, BCL6, MUM1, MYC, BCL2, FOXP1, and IgM as described previously^15,16^.

### Sequencing and copy number variations (CNV) detection

At the time of diagnosis, gDNA and cfDNA were sequenced with the entire Lymphopanel. Ion Torrent Personal Genome Machine (Thermo Fisher Scientific) Sequencing was performed using our Lymphopanel as previously described. Briefly, the Lymphopanel was designed to identify mutations in 34 genes important for lymphomagenesis grouped into 8 specific pathways. This design covers 87,703 bases and generates 872 amplicons. Variant analysis was performed using an in-house generated bioinformatics pipeline that was previously described^6,13,17^. CNV detection was performed using ONCOCNV software as previously described^17,18^.

Sanger sequencing was also performed to validate a portion of the mutations found in tumoral gDNA using the BigDye^®^ Terminator v3.1 Cycle Sequencing Kit and an ABI PRISM 3130 analyzer (Applied Biosystems, Forster City, CA, USA).

During follow-up, to increase the sensitivity and for cost-effectiveness of the sequencing, only mutated amplicons detected at baseline were sequenced and used to detect minimal residual disease in cfDNA.

The tumoral cfDNA (ctDNA) concentrations were expressed in haploid genome equivalents per mL of plasma (hGE/mL) and calculated by multiplying the mean variant allelic frequency (VAF) by the concentration of cfDNA (pg/mL of plasma) and dividing by 3.3, as previously described in the publication by Scherer et al^12^.

### PET scan analysis

All patients underwent [^18^F]fluorodeoxyglucose-PET/CT (FDG-PET/CT) before the onset of chemotherapy in the nuclear medicine department, according to standard procedures. The following parameters were determined on the baseline scan: (1) SUVmax, the highest SUVmax measured in the tumor sites and (2) total metabolic tumor volume (MTV), which was obtained by summing the metabolic volumes of all the nodal and extranodal lesions, a volume of interest was set around each lesion (node or organ involvement) as previously described^19^. The bone marrow involvement was only included in the volume measurement if there was focal uptake. The spleen was considered as involved if there was focal uptake or diffuse uptake higher than 150% of the liver background. Total lesion glycolysis (TLG) was also calculated as the sum of the product of the metabolic volume of each local tumor based on its SUVmean (TLG = ΣMTV × SUVmean). For segmentation, the 41% SUVmax threshold method was used^19,20^. iPET was planned in all treated patients at mid-treatment, allowing us to assess early response using the 5-point Deauville score (5-DS) and the DeltaSUV (ΔSUVmax) calculation as previously reported^21^. PET was also performed at the end of treatment and the response was assessed according to the current recommendations (Supplementary Fig. S1)^22^.

### Statistical analysis

All statistical analyses were performed using R software version 3.1.2^23^. Progression-free survival (PFS) was evaluated from the date of enrollment to the date of disease progression, relapse or death from any cause. Overall survival (OS) was evaluated from the date of enrollment to the date of death from any cause. Log-rank tests were used to assess differences in the OS and PFS rates calculated by Kaplan–Meier estimates. Statistical differences between parameters in box plots were determined using the Wilcoxon test. *P*-values < 0.05 were considered statistically significant.

## Results

### Patient population description

A prospective series of 30 consecutive untreated DLBCL or PMBL patients were enrolled and followed after R-CHOP or R-CHOP-like chemotherapies. Patients’ clinical features are listed in Table 1. One patient died before beginning any chemotherapy. From mid-treatment, 17 patients achieved complete response (Deauville score ≤ 3), 2 were stable (absence of metabolic response), and 10 showed a partial response (Deauville score > 3). At the end of treatment, 1 more patient had died, 6 patients presented a partial response, 4 a progression, and 18 a complete response. At the end of the trial, 1 patient decided to stop follow-up, 20 patients showed a complete response, 3 relapses were observed including 1 with premature death, 2 progressive patients had died, and the 2 others still presented a progression (Supplementary Fig. S2, Supplementary Table S2).

**Table 1:** Patients characteristics

Characteristics	Patients (%)
Men/women	17/13
Median age	67 [20–93]
IPI score	
0–1	8 (27)
2–3	14 (46)
4–5	8 (27)
LDH > normal value	12 (40)
Stade III–IV	21 (70)
Bone marrow involvement	0/25 (0)
COO classification	
Hans (Non-GCB/GCB/NA)	9 (34)/17 (66)/5
MYC + expression	7/25 (28)
BCL2 + expression	16/24 (66)
Dual expression	5/21 (24)
First-line treatment	
RCHOP	16 (55)
RCHOP-like regimen (RACVBP/R miniCHOP)	13 (45)
Treatment response	
CR	20
PR	5
PD	1
TEP base line features	
SUV max	22.61 [4.66–43.03]
Metabolic tumor volume (MTV)	399.28 [0.29–2846]
Total lesion glycolysis (TLG)	4697.50 [1.17–31149]
Delta SUV at interim PET	
>70%	22
<70%	7
Deauville score at interim PET	
1–3	17
4–5	12
Deauville score at final PET	
1–3	14
4–5	10

### Plasma genotyping at baseline

**Fig. 1:**
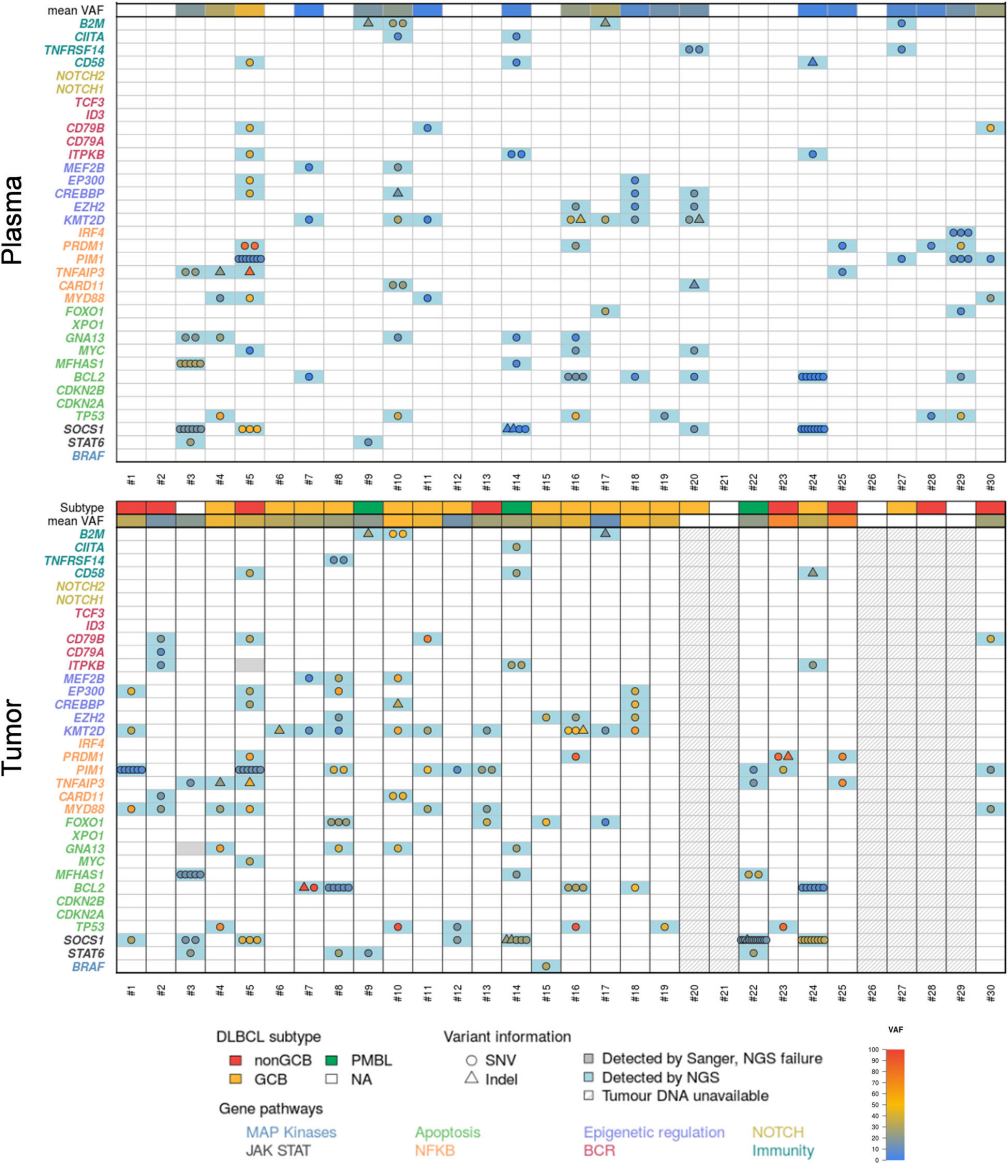
Basal genetic patterns in plasma cfDNA and matched tumoral DNA. Representation of the prevalence and molecular spectrum of somatic mutations (SNVs and Indels) identified in cfDNA (upper panel) and paired tumoral DNA (lower panel) at the time of diagnosis. The 34 genes in the Lymphopanel are grouped by pathway

To determine the clinical relevance of liquid biopsy at the time of diagnosis, we first assessed the basal DLBCL genetic patterns with cfDNA by targeted NGS. CfDNA was available from the 30 patients. The mean plasma DNA concentration was 74 ng/mL [14.6–433], and sequencing was performed with the entire panel with a mean depth of 3750×. cfDNA mutations were identified in 19/30 patients (63%). The mutation profiles were consistent with patterns usually observed in DLBCL and previously reported by analyzing the tumor tissues^13,17^ (Fig. 1).

*KMT2D* (*MLL2*) and *PIM1* were the most mutated genes, with somatic mutations in 36.7% of cases (*n* = 11/30), followed by *SOCS1* and *TP53* in 26.7% (*n* = 8/30); *MYD88* and *BCL2* in 23.3% (*n* = 7/30); *GNA13* and *PRDM1* in 20% (*n* = 6/30); *EZH2*, *FOXO1*, and *TNFAIP3* in 16.7% (*n* = 5/30); *ITPKB*, *CD79B*, *B2M*, *STAT6*, *EP300*, and *CREBBP* in 13.3% (*n* = 4/30); *CARD11*, *MFHAS1*, *TNFRSF14*, *MEF2B*, *CD58*, and *MYC* in 10% (*n* = 3/30); *CIITA* in 6.7% (*n* = 2/30); and *IRF4*, *CD79A*, and *BRAF* in 3.3% (*n* = 1/30). The mutation distribution was associated with the COO subtype, such as *MYD88* (particularly L265P), *PIM1*, and *CD79B* in ABC DLBCL; *EZH2*, *BCL2*, and *GNA13* in GCB DLBCL or *STAT6*, *SOCS1*, and *MFHAS1* in PMBL. Multiple mutations were identified in genes such as *IRF4*, *SOCS1*, *PIM1*, and *BLC2*, suggesting an AID process as previously described^13^.

The cfDNA VAF was very variable with a mean of 18.2% [0.8–87.4]. The mean concentration of ctDNA was 4604 hGE/mL [0–39,151]. A high concentration of cfDNA signaled the presence of circulating DNA from tumoral origins, as no plasma samples with cfDNA concentrations above the average were negative for mutations (*p* = 5.6 × 10^−4^, Supplementary Fig. S3).

### Tumor genotyping at baseline and concordance with plasma genotyping

When available, matched gDNA from tissue biopsies was sequenced with the Lymphopanel and compared to the cfDNA results. Enough material was obtained for 25 patients, including 11 frozen and 14 FFPE biopsies.

The sequencing results were interpretable for 24 cases and not interpretable for 1 case (#28) (Fig. 1). Mutations were found in the 24 interpretable cases; for the 6 remaining cases without workable tumoral materials, an informative mutational pattern was successfully obtained in 4 matched cfDNA. Finally, the mutations at baseline were detected either in gDNA or ctDNA in 28/30 cases, giving an informativity rate of 93%.

In five cases, additional mutations were only detected in cfDNA compared to gDNA (#3, #5, #10, and #16). Conversely, some mutations found in gDNA were not found in cfDNA, as it has been previously described^6,7^. In these cases, the mutations did not necessarily have the lowest VAF in the gDNA, showing that the mutations were not subclonal. For paired samples, mutations were detected in cfDNA up to a VAF of 4.6% in gDNA (patient #7). The mean VAF was significantly higher in tumor DNA (33%) compared to cfDNA (*p* = 5.6 × 10^−7^), but in some cases the VAF in cfDNA was nearly similar to the VAF in gDNA, reflecting the abundance of circulating DNA from tumoral origins.

After sequencing the tumors, we confirmed the results in cfDNA by performing a new sequencing method targeting only the identified mutations to increase sensitivity (Supplementary Table).

### CNV analysis at baseline

CNV analysis was performed in both plasma and tumor samples using the entire panel. As expected in DLBCL cases, we frequently found deletions at the 6q and 9p loci (33% and 37%, respectively) as well as *TP53* deletions (13%) in the DLBCL cases. The gains and/or losses identified were similar in the paired samples from a patient. Discordances were principally due to the quantity of tumoral DNA contained in the sample from the tissue biopsy or plasma (according to mean VAF, Supplementary Fig. S4).

**Fig. 2:**
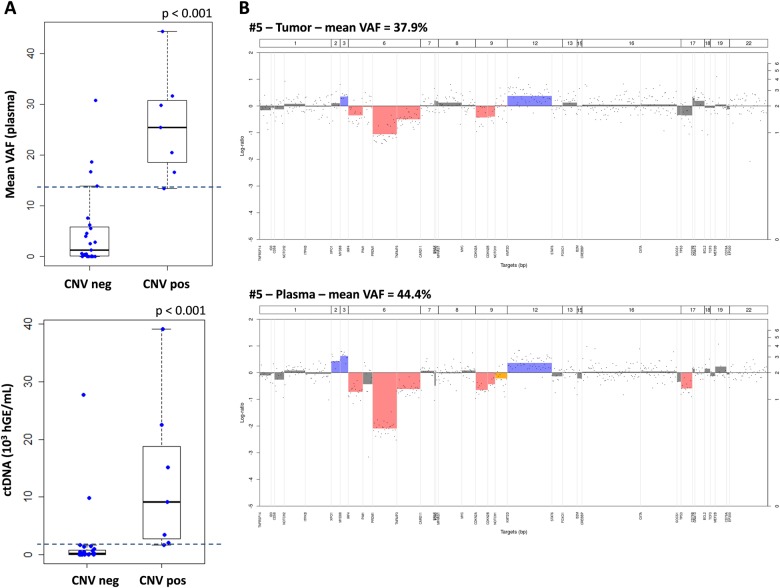
CNV analysis in plasma and tumor. **a** Boxplot representation of mean VAF (upper panel) or ctDNA amount (lower panel) according to the detection of the CNVs in the plasma samples (neg = negative and pos = positive). The dashed blue lines show the thresholds of detection for CNVs in cfDNA, corresponding to 13.4% for the mean VAF and 1630 hGE/mL for ctDNA. **b** Example of CNVs from patient #5 in tumor (upper panel) and plasma (lower panel) showing the concordance of the two results when cfDNA of tumoral origin is abundant

In cfDNAs, CNVs were detectable when tumoral circulating DNA was abundant; according to the mean VAF from the seven positive patients, this was when the tumoral circulating DNA ranged from 13 to 45% (*p* < 0.001, Fig. 2, Fig. S4). Above this threshold, patients negative for CNVs in cfDNA were also negative in tumoral samples, except for one patient (#19). For this particular case, the mean VAF was 14%, but with only one mutation, while patient #29 had a mean VAF of 13.4% calculated from 10 mutations with frequencies between 6 and 40%. Therefore, with a threshold of 13.4% for mean VAF in the cfDNA, CNVs were detectable in 91% of the cases (10/11). If we expressed these results based on the quantity of ctDNA using a threshold of 1630 hGE/mL for ctDNA, all the positive plasmas were above this value, highlighting the importance of both the VAF and cfDNA concentration at the time of diagnosis.

### Correlation between cfDNA and clinical or TEP baseline features

**Fig. 3:**
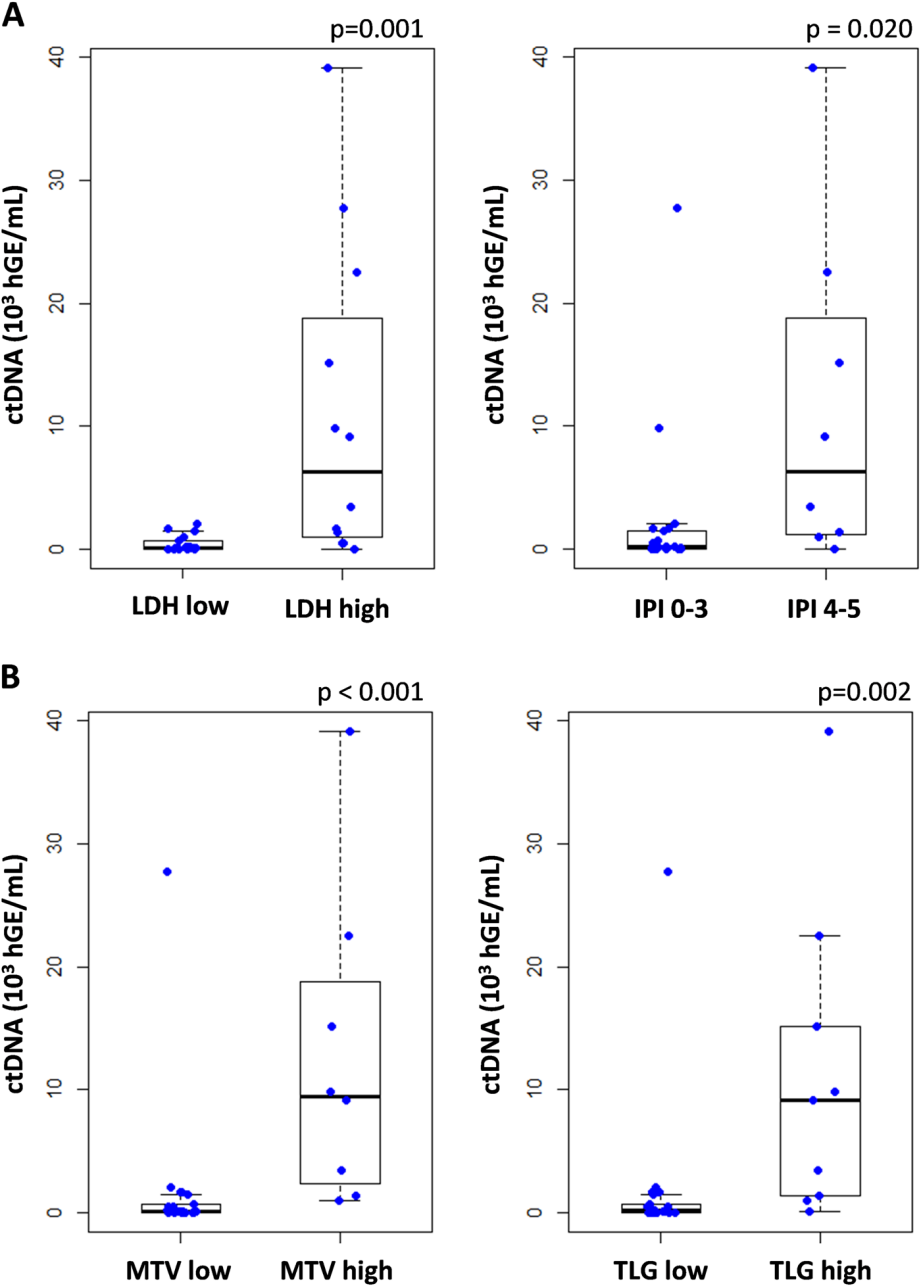
Correlations between tumoral cfDNA amounts and clinical/PET baseline features. CtDNA amount is significantly correlated with the clinical indices LDH (threshold = 480 UI/L) and IPI (**a**), and with the PET features MTV and TLG (threshold = mean) at the time of diagnosis (**b**)

We first compared the presence of ctDNA with standard clinical indices. CfDNA mutations were observed in all patients but one with elevated serum lactate dehydrogenase (LDH), but the presence of mutations was not correlated to the disease stage. The mean VAF in cfDNA, as well as cfDNA concentration, were significantly correlated with LDH and the IPI. The quantity of ctDNA was more significantly correlated with these two features (*p* = 0.001 and *p* = 0.02, respectively, Fig. 3a), once again highlighting the importance of both the VAF and cfDNA concentration at the time of diagnosis.

PET scan imaging (^18^F-FDG PET/CT) data were collected for all the patients at baseline. The mean MTV was 743.69 cm^3^ (0.29–2846.62) and the mean TLG was 8341.96 (1.17–31149.00). At the time of diagnosis, both MTV and TLG correlated strongly with the ctDNA concentration (*p* < 0.001 and *p* = 0.002, respectively, Fig. 3b), and more significantly than the concentration or VAF alone, as for clinical features. Patients with an MTV below the median (399.28 cm^3^) had a superior OS and PFS compared with those with a high MTV (*p* = 0.016 and *p* = 0.009, respectively, Fig. S5).

**Fig. 4:**
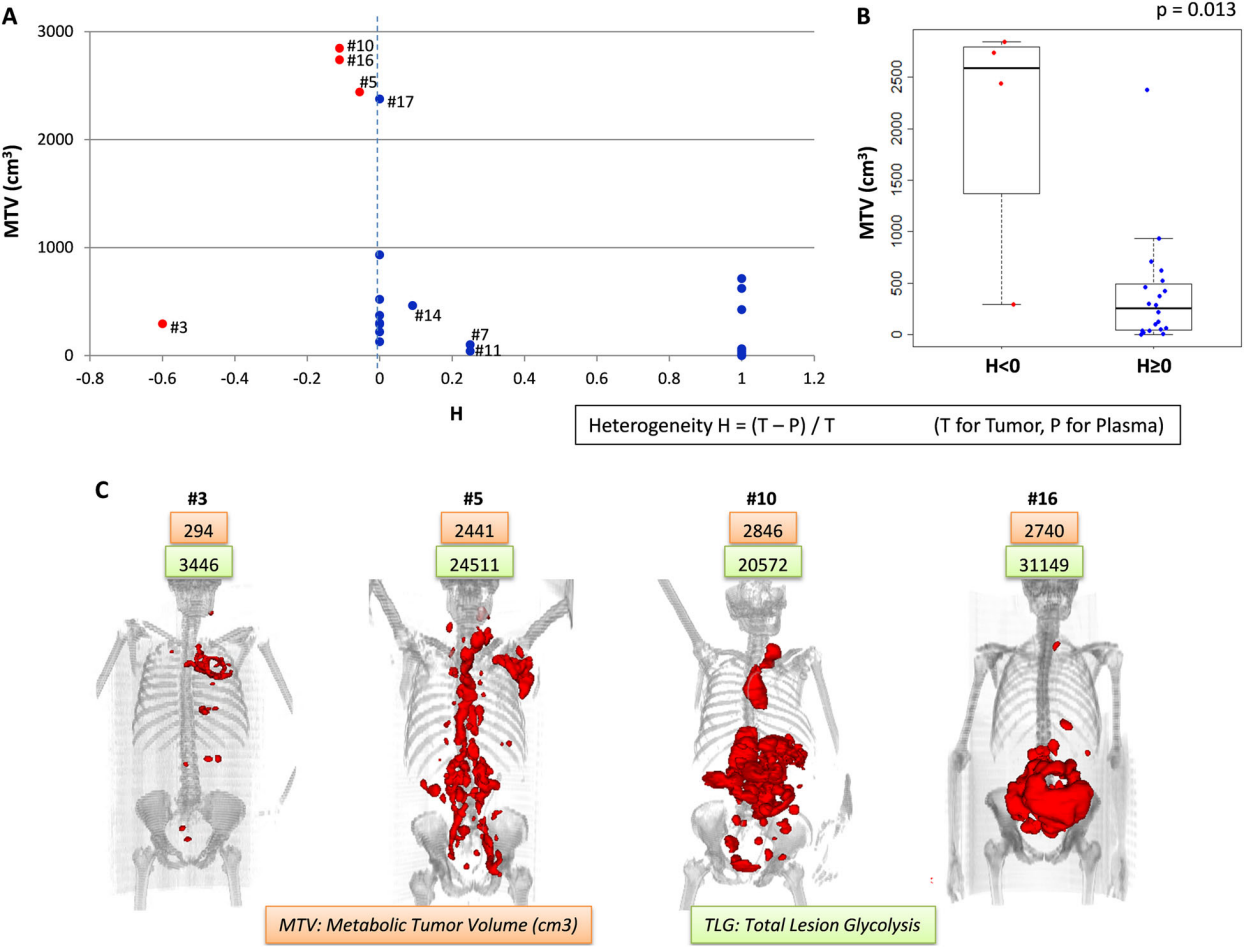
Tumoral heterogeneity. **a** Metabolic tumor volume according to the coefficient of heterogeneity (H = (Number of mutations in tumor DNA−Number of mutations in cfDNA)/Number of mutations in tumor DNA). The red dots represent the patients with more mutations detected in the cfDNA than in the tumoral DNA (H < 0). **b** Boxplot representation of the MTV values according to the presence (red dots, H < 0) or absence (blue dots, H ≥ 0) of supplementary mutations in the cfDNA. **c** 3D view of PET scan images for the four patients presenting supplementary mutations in the cfDNA, with MTV in orange boxes and TLG in green boxes

We calculated a heterogeneity coefficient H representing the number of mutations found in the cfDNA compared to gDNA (H = (Number of mutations in gDNA−Number of mutations in cfDNA)/Number of mutations in gDNA). This coefficient was significantly correlated with the MTV (*p* = 0.013, Fig. 4b). Three in four patients (#5, #10, and #16) presenting additional mutations in the cfDNA, compared to gDNA (*h* < 0), had a high MTV above 2000 cm^3^ (Fig. 4a). The 3D view of the PET images showed that two of these patients had many tumoral masses (#5 and #10), as well as a fourth patient who had a low MTV despite additional mutations in its cfDNA (#3, Fig. 4c). These data suggest that cfDNA mutation analysis more accurately reflects the spatial tumor heterogeneity than tissue biopsy analysis. Conversely, the three patients (#7, #11, and #14) with more mutations in tumor DNA (0 < *h* < 1) had lower MTV values (<500 cm^3^). Interestingly, patient #17 presented the same mutations in its cfDNA and tumor DNA (*h* = 0) and an important MTV (2375 cm^3^), but the mutation VAFs were high in the plasma sample compared with the tumor sample. All the patients with negative plasma (*h* = 1) had MTVs less than 800 cm^3^.

### CfDNA monitoring and correlation with iPET and final analysis

Longitudinal analysis of the plasma samples collected at mid-treatment and at the end of treatment or at relapse/progression was performed and correlated with the PET scan characteristics (ΔSUVmax and Deauville score).

**Fig. 5:**
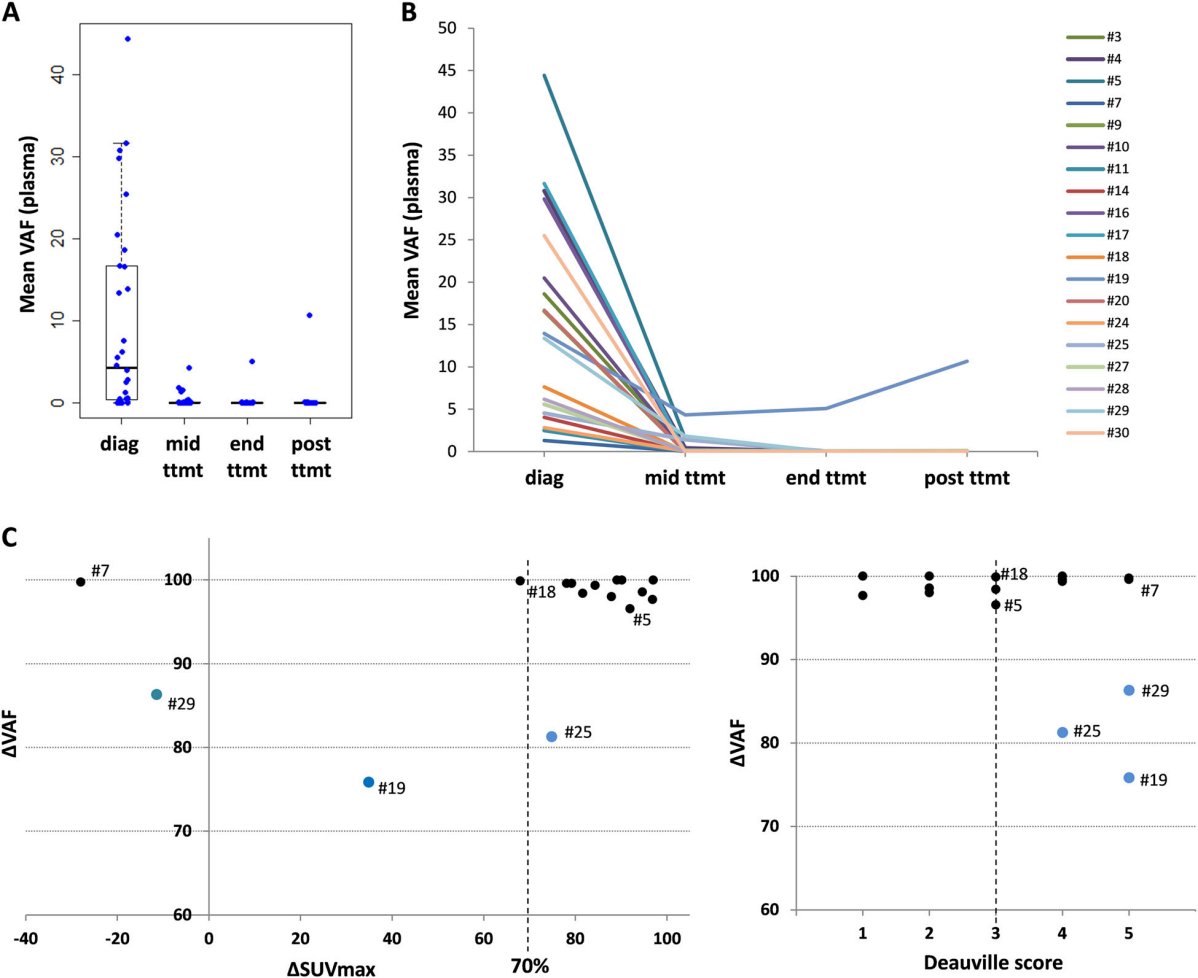
Longitudinal assessment of mutation abundance in plasma cfDNA upon R-CHOP treatment according to interim PET scan. **a** Distribution of the cfDNA mean VAFs of the patients at the different times of follow-up (diagnosis, mid-treatment, end of treatment, and 6 months post-treatment). **b** Evolution of the cfDNA mean VAFs for each patient throughout treatment. **c** ΔVAF values in plasma according to the ΔSUVmax (left) or Deauville score (right) between diagnosis and mid-treatment. The vertical dashed lines represent the cut-off ΔSUVmax of 70% (left) or Deauville score of 3 (right), and the blue dots represent patients with ΔVAF <90%

Upon treatment with R-CHOP/R-CHOP-like regimens, a rapid clearance of cfDNA mutations was observed in the 16 baseline positive cases available at mid-treatment (Fig. 5 and Supplementary Table S1). In four cases (#5, #19, #25, and #29), the basal DLBCL mutations did not completely disappear at mid-treatment. Among these four patients, three had a delta VAF (ΔVAF) less than 90% and were in partial response according to their Deauville Scores (#19, #25, and #29), including two with a ΔSUVmax <70% (#19 and #29). Patient (#25) had a ΔSUVmax of 75%, though the ΔVAF was 81% and ctDNA was still detectable (VAF = 1.4%). Moreover, patient #29 finally showed progression and died before the end of treatment. Conversely, for patient #7, the mutations disappeared from the cfDNA at mid-treatment (ΔVAF near 100%) despite the absence of metabolic response (Deauville score of 5) and a ΔSUVmax <70% (−28).

Unfortunately, at the end of treatment, some important plasma samples were missing (only 20 available). Patient #10, who presented progressive disease, had no plasma samples either at the end of treatment or after. Patient #25 had a partial response and circulating DNA of tumoral origin at mid-treatment, but no plasma samples were collected for further follow-up.

### Patients’ examples

**Fig. 6:**
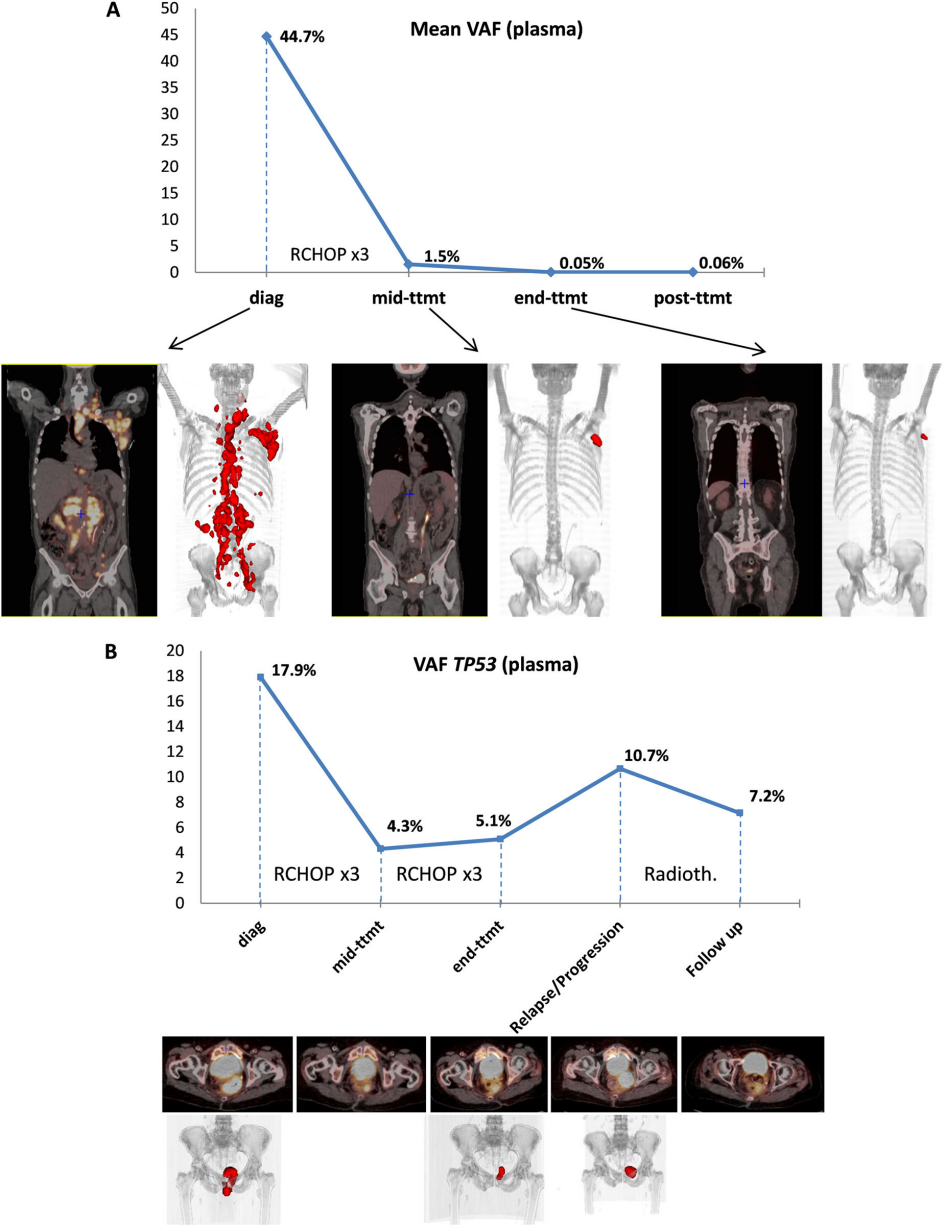
Examples of non-invasive real-time monitoring of the DLBCL clonal evolution in plasma cfDNA. Mean VAF and PET scan images at different times during follow-up for patient #5 (**a**) and patient #19 (**b**)

As an example, two emblematic patients were more extensively described (Fig. 6).

Patient #5 had one of the highest MTVs in its baseline PET scan (2441 cm^3^), with an important dissemination of the tumoral mass showed by the 3D view of the PET images (Figs. 4c, 6a). The plasma sample was very rich in tumoral cfDNA according to the important VAF values, which had a mean of 44.4% (ctDNA amount of 22,508 hGE/mL), and the CNV analysis showed the same gains and deletions in the plasma and tumor (Fig. 2b). More mutations were observed in the cfDNA compared with tumoral DNA (Fig. 4 and Supplementary Table S1), indicating spatial tumor heterogeneity that was probably correlated with the dissemination observed in the PET images. At mid-treatment, this patient showed a complete response with a Deauville score of 3 and a ΔSUVmax of 92%. The ΔVAF was 97%, but we still detected tumoral DNA circulating in the plasma with a mean mutation VAF of 1.5%. At the end of treatment and 6 months post treatment, this patient had yet a complete response and no more tumoral circulating DNA was found. Despite an unfavorable somatic mutation profile, including *MYD88* mutations and a *CDKN2A* deletion, the patient is still considered in CR after 2 years of follow-up.

Patient #19 had a *TP53* mutation detected both in its cfDNA (VAF = 14%) and tumoral gDNA (Supplementary Table S1). The plasma sample was informative despite the low MTV (128 cm^3^). This mutation was still present in the cfDNA at mid-treatment, but with a decreased VAF (4.3%, ΔVAF = 75%), though the patient was partially responding to treatment with a ΔSUVmax of 35% and a Deauville score of 5. Of note, even if we observed a decrease in the *TP53* mutation frequency, the ΔVAF of 75% was the lowest ΔVAF in the cohort. At the end of treatment, the patient was still in partial response according to the PET scan images and still harbored tumoral cfDNA, as shown by a persistent detectable *TP53* mutation with a VAF of 5% (Fig. 6b). Four months after the end of treatment, the patient had progressive disease and the frequency of the *TP53* mutation increased to 10.7%. Five months later, treatment by radiotherapy led to an improvement according to PET scan data, though the two following PET scans showed progression and a stable persistent hypermetabolism that was not specific. Finally, we analyzed an extra plasma sample (nearly 2 years after the end of treatment) and observed that the same *TP53* mutation was still present at a VAF of 7.16%, supporting the better sensitivity of the liquid biopsy.

## Discussion

In this prospective study we assessed basal DLBCL genetic patterns, their modification upon treatment using plasma cfDNA analysis, and their correlation with PET scan imaging by NGS. In complement with previously published results obtained from tumor biopsies from patients enrolled in LYSA clinical trials, we demonstrated here that the 34-gene Lymphopanel (87,703 bp) is also a reliable genotyping tool to characterize tumoral cfDNA in plasma^13,17^. CfDNA mutations were identified in 19/30 patients (63%) cases, a similar rate to that reported by Rossi et al.^7^ in the training cohort (2/30, 66%). In the validation cohort, 17/20 (85%) DLBCL patients harbored cfDNA mutations, a higher rate that can be explained by another distinct technology (CAPP-Seq) and a larger gene panel (59 genes, 207,299 bp). However, despite the quantitative and qualitative differences of the interrogated genes, CAPP-Seq and the Lymphopanel-Ampliseq-based technologies allowed the recovery of at least one clonal mutation in 92.6 and 96% of DLBCL patients, respectively, as documented by in silico validation or cohort sequencing^7,13^. This suggests that the discrepancy observed between the two studies is most likely related to the higher sensitivity of the CAPP-Seq method. Nevertheless, we identified mutations in cfDNA up to a VAF of 4.6% in gDNA, while in the Rossi et al. study the cut-off for detection was 20%. Of note, we also recently tested our Lymphopanel-Ampliseq approach in PCNSL and showed that it represents a reliable genotyping tool in a setting where tumor cfDNA amounts are supposed to be low and inconstant^8^. For these reasons, we are currently developing new approaches of sequencing, as an alternative of CAPP-Seq, to date not widely implemented in routine, using our lymphopanel with molecular barecoding and dedicated bio informatic tools in order to improve sensitivity.

The 34-gene Lymphopanel has been extensively performed in more than 300 DLBCL cases^17^ and was initially design to discriminate the three main molecular subtypes, namely, the GCB, ABC, and PMBL subtypes and to provide markers for personalized medicine and targeted treatment. The results obtained by cfDNA genotyping are consistent with the patterns usually observed in the different subtypes and confirm the clinical relevance of the panel to detect targetable variants such as *EZH2*, *CD79B*, or *MYD88* hotspot mutations. Of note, three among the four PMBL cases were informative in both tumor and plasma and displayed a typical genotype, suggesting that cfDNA is likely a promising tool for the management of PMBL.

Importantly, we were also able to detect CNVs that accurately and specifically matched with gDNA in some cases (Fig. 2), including the unfavorable prognostic deletion of *TP53* or *CDKN2A*. However, in our hands and with the AmpliSeq Technology, CNVs were detectable in cfDNA in a limited number of cases, with a minimal mean VAF of 13% or 1630 hGE/mL ctDNA.

Factors that influence the amount of tumor cfDNA are partially known. Here, we confirmed previous works showing that baseline cfDNA amounts strongly correlate with tumor burden indicators including LDH level and the IPI score but also MTV or TLG^12^. Moreover, the patients presenting additional mutations in cfDNA, compared to gDNA, had a high MTV, suggesting that cfDNA mutations more accurately reflect tumor heterogeneity than gDNA analysis, which is limited by the unique biopsy site. Regarding the well-established prognostic value of the MTV in DLBCL, cfDNA amounts may explain the strong prognostic values recently reported in DLBCL^12,24^. In addition to the combined analysis of the MTV and GCB/ABC status or BCL2/MYC expression^25^, it appears relevant to obtain both cfDNA and MTV at baseline to risk stratify newly diagnosed DLBCL.

We performed longitudinal analysis of plasma samples collected at mid-treatment, at the end of treatment, or at relapse/progression and correlated the results with PET scan characteristics. A rapid decrease in the cfDNA mutation VAF was observed in most cases. Rapid clearance of ctDNA after two cycles of R-CHOP, higher than 2 log, has been recently reported as a strong prognostic marker^24^. From mid-treatment, to increase the sequencing sensitivity and for cost-effectiveness reasons, only mutations detected at baseline were monitored during the follow-up. Some patients with partial remission at mid-treatment, according to their Deauville score and ΔSUVmax (<70%), still had basal DLBCL mutations circulating in the plasma. Conversely, despite the partial response, some patients had no ctDNA in the plasma. Thus, the targeted approach is questionable because it precludes any detection of new (or subclonal) variants emerging during treatment, as it has been reported by Rossi et al^7^. Regardless of the liquid biopsy value, follow-up with the entire panel is recommended. These data also suggest that interim iPET and cfDNA can both be used to define early response during DLBCL treatment^9^. Their respective and complementary relevance remains to be determined in a larger cohort.

Interestingly, one elderly patient (#19) in partial remission had progressive disease 6 months after the end of treatment and still harbored tumoral cfDNA, as shown by a persistent detectable pathogenic *TP53* mutation. However, the tumor volume still remains stable and, importantly, the mutation was also detected in the bone marrow without any evidence of invasion (according to PET scan and bone marrow sample analysis). Persistent variants should be analyzed with caution, especially those targeting *TP53* in elderly patients because it has been demonstrated that blood cells from more than 2% of individuals (5–6% of people older than 70 years) contain mutations that may represent premalignant events that cause clonal hematopoietic expansion^26,27^. Age-associated, low-frequency TP53 mutations were also found in 100% of peripheral blood samples from 15 women with and without ovarian cancer (none with hematological disorder)^28^.

To conclude, our prospective study demonstrates that cfDNA genotyping of DLBCL is an accurate genotyping tool and represents a real-time and non-invasive approach for follow-up. It highlights the major interest of liquid biopsy in the context of bulky tumors, where cfDNAs are more representative than gDNA for capturing the entire tumoral mutation profile. Therefore, cfDNA analysis represents a complementary approach to PET scan imaging at baseline and during follow-up for the management of DLBCL.

## Electronic supplementary material

Supplementary figures

Supplementary Table S1: Mutations and VAF in tumors and plasmas at diagnosis and during follow-up

Supplementary Table S2: Patients’ data
