# Supplementary material for: Non-invasive monitoring of diffuse large B-cell lymphoma by cell-free DNA high-throughput targeted sequencing: analysis of a prospective cohort
Source: Blood Cancer J. 2018 Aug 1;8(8):74. doi: 10.1038/s41408-018-0111-6 (PMC6070497; doi:10.1038/s41408-018-0111-6)
Supplement: Supplementary file 1 — Supplementary figures [file 41408_2018_111_MOESM1_ESM.pdf]

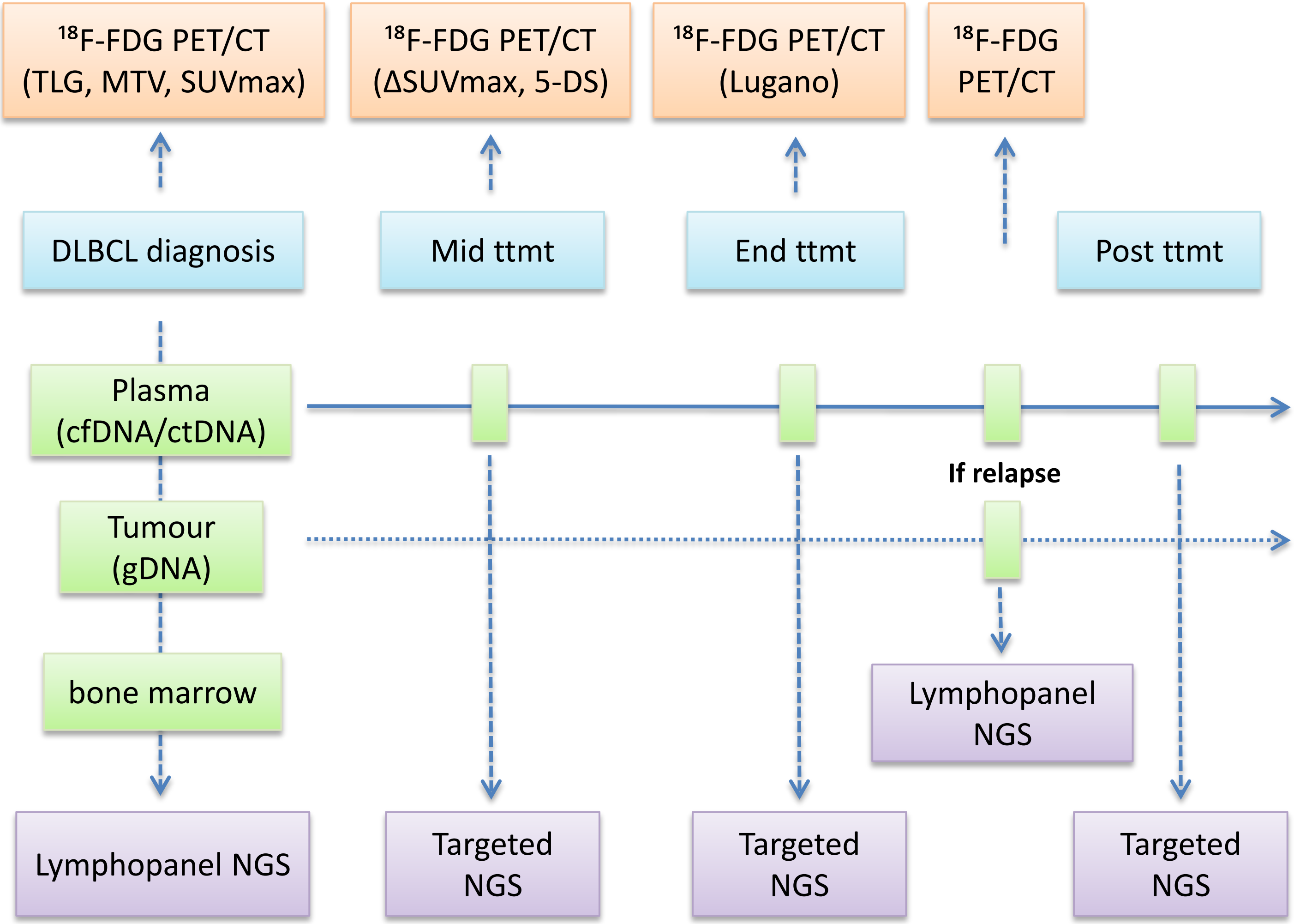

Figure S1: Process flow chart.

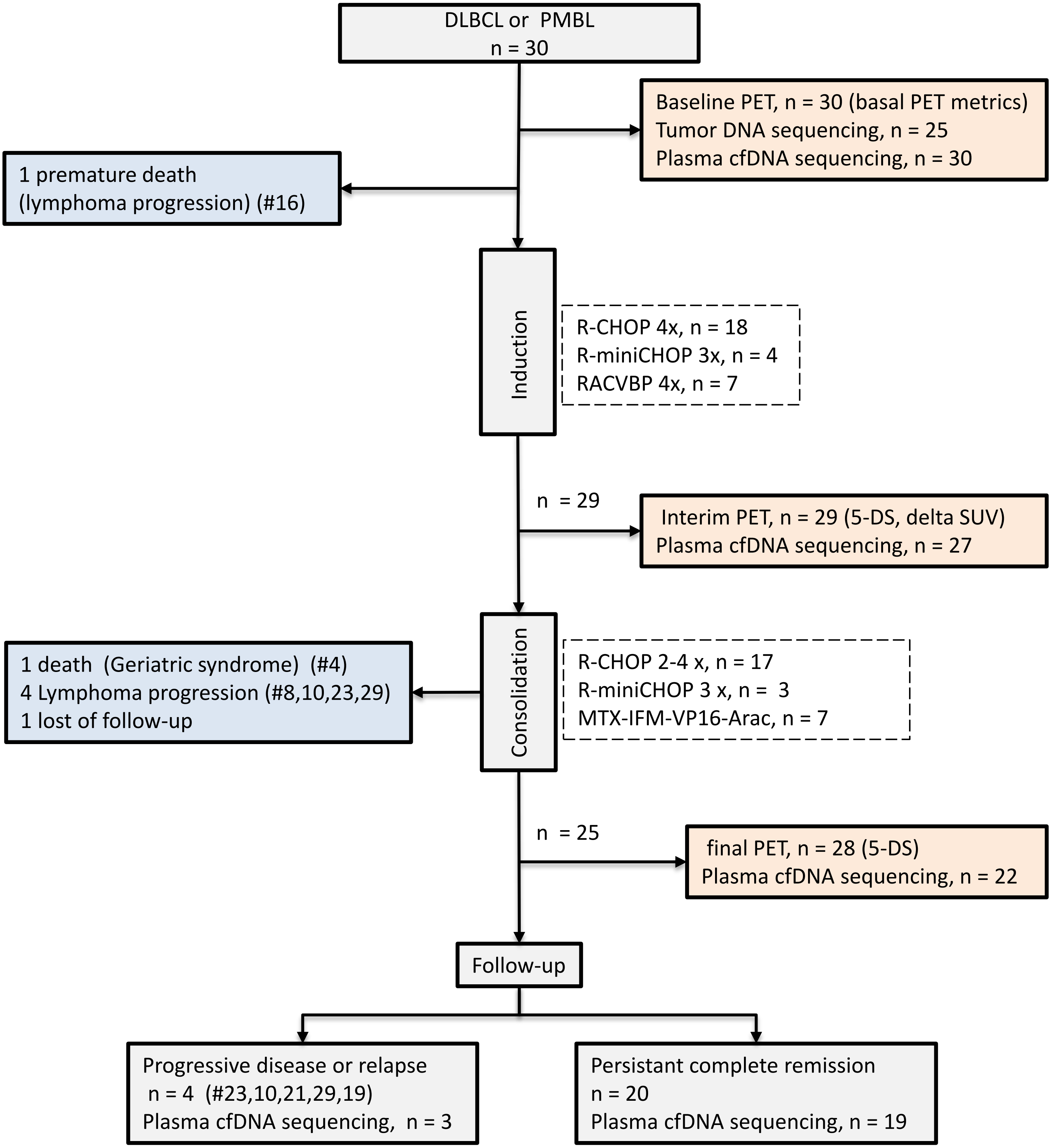

Figure S2: Analysis flow chart.

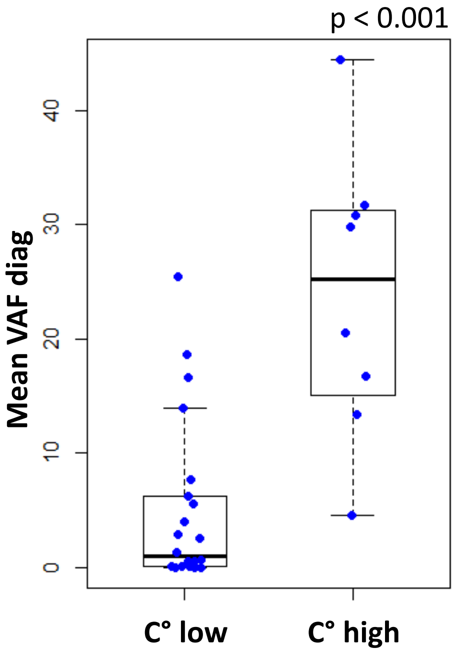

Figure S3: Correlations between mean VAF in cfDNA and concentration of cfDNA at baseline.
(cut off for concentration low/high is mean concentration)

Patient #1 (tumor-VAF = 33.7% / plasma-VAF = 0.06%)

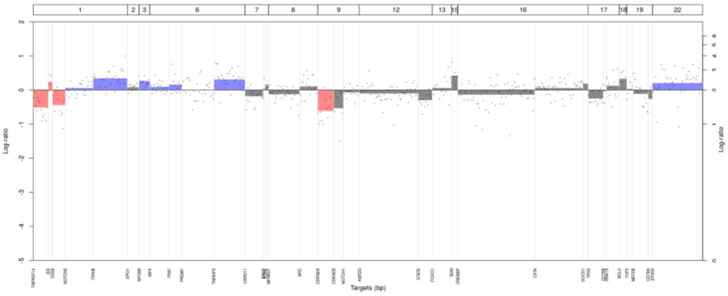

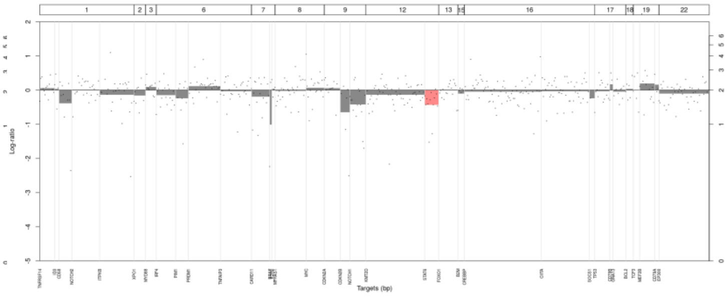

Patient #2 (tumor-VAF = 16% / plasma-VAF = 0.13%)

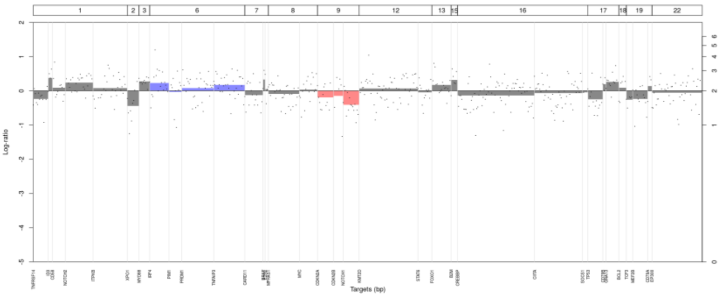

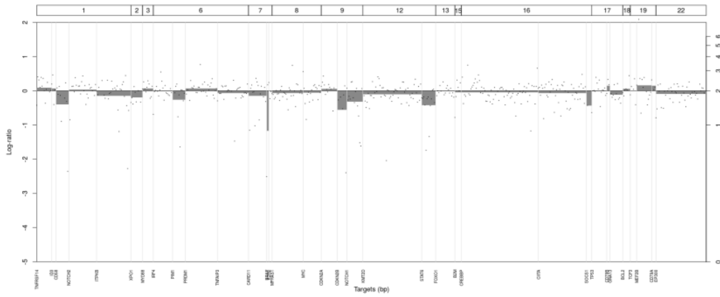

Patient #3 (tumor-VAF = 20.5% / plasma-VAF = 18.6%)

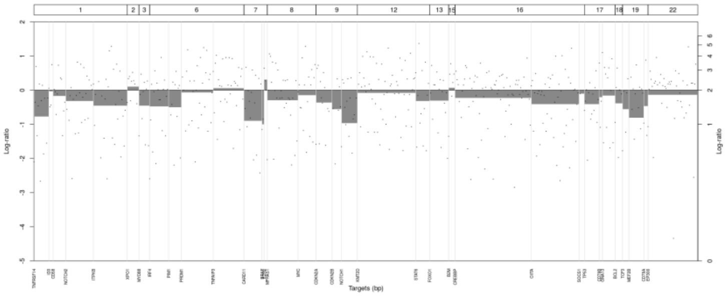

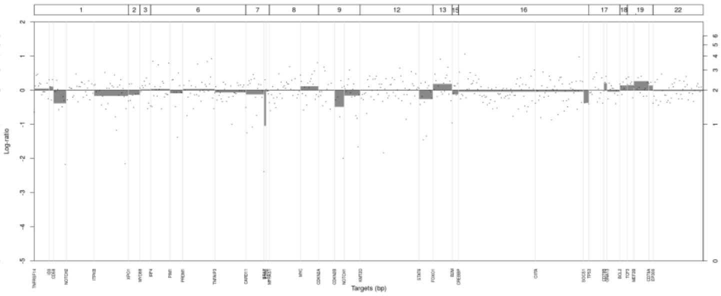

Patient #4 (tumor-VAF = 45.8% / plasma-VAF = 30.8%)

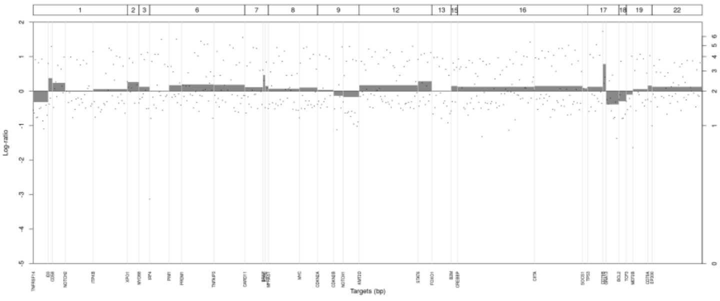

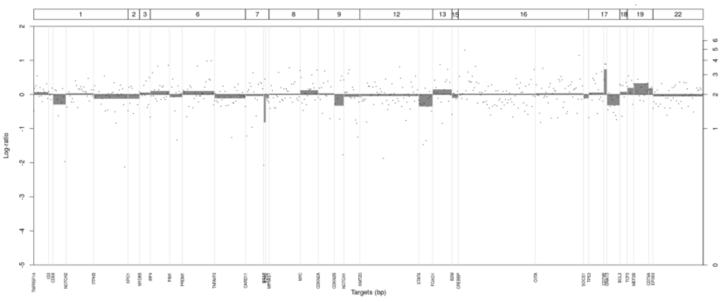

Patient #5 (tumor-VAF = 37.9%/ plasma-VAF = 44.4%)

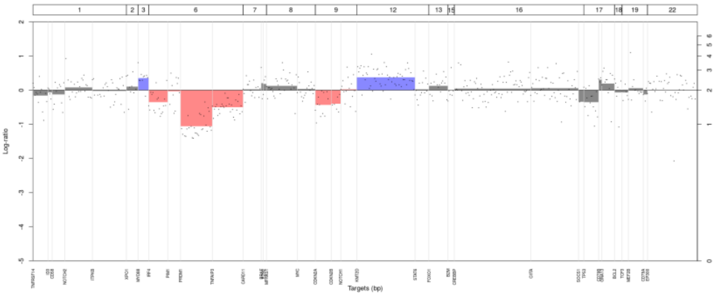

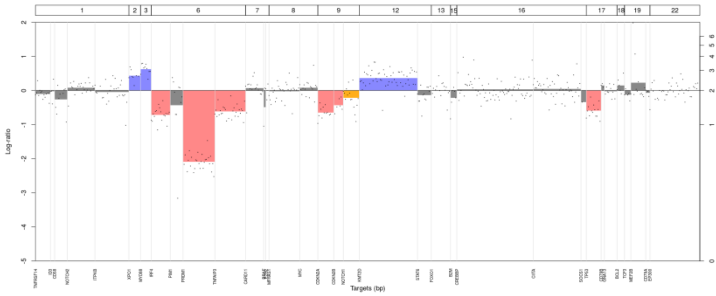

Patient #6 (tumor-VAF = 32.3% / plasma-VAF = 0%)

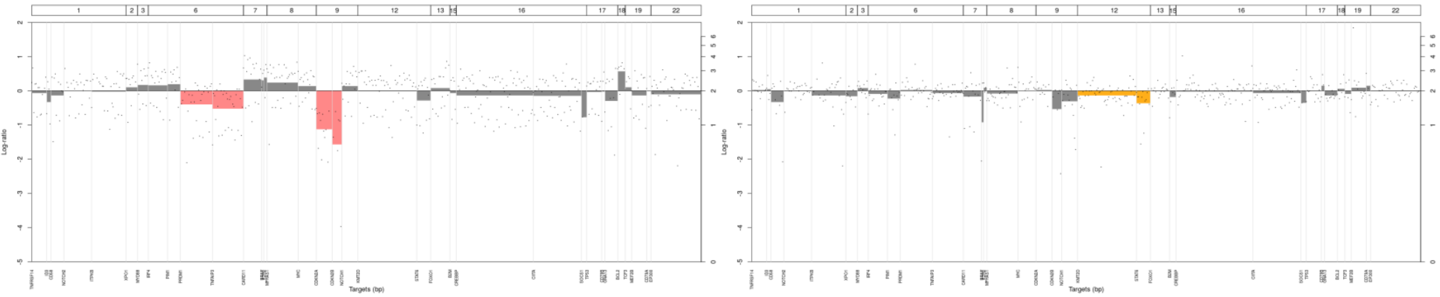

Patient #7 (tumor-VAF = 28.3% / plasma-VAF = 1.3%)

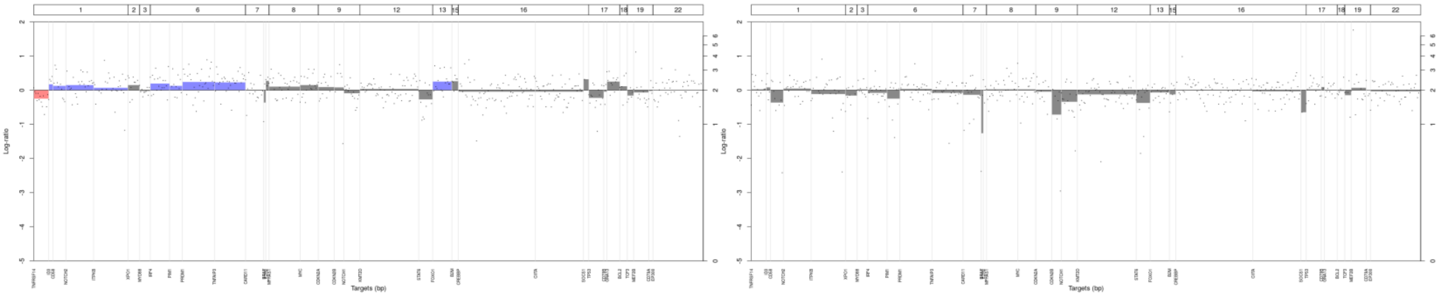

Patient #8 (tumor-VAF = 25.8% / plasma-VAF = 0.54%)

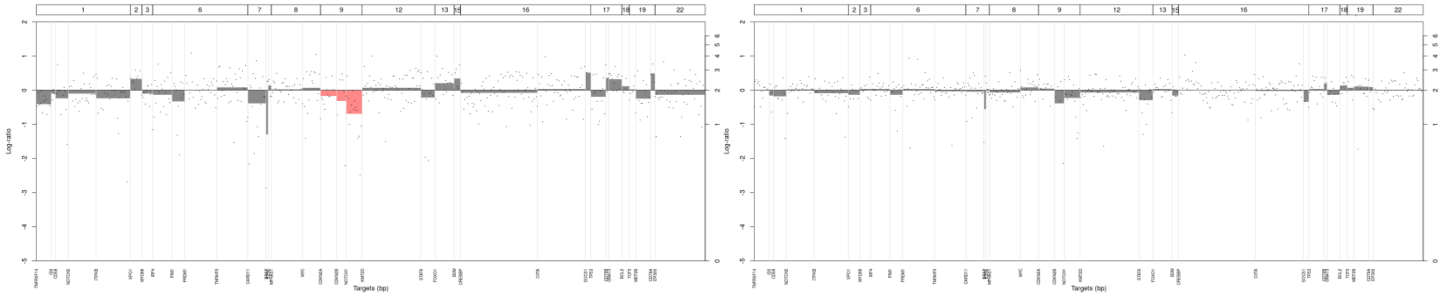

Patient #9 (tumor-VAF = 21.3% / plasma-VAF = 16.6%)

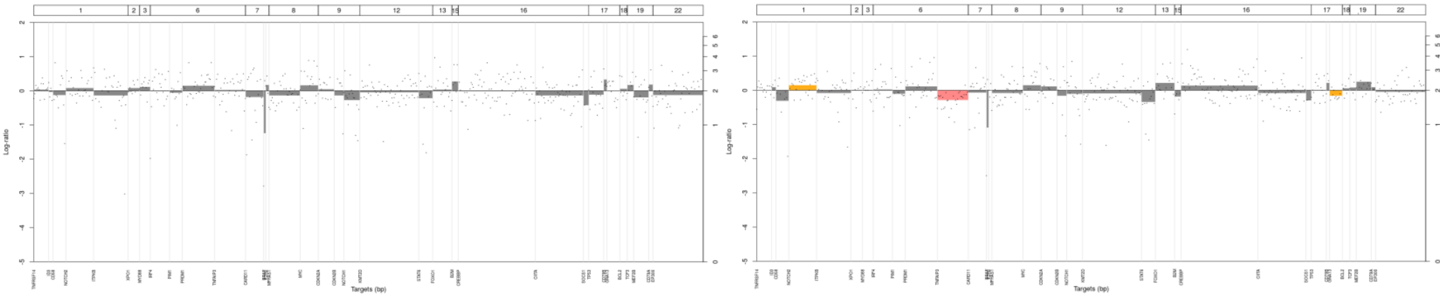

Patient #10 (tumor-VAF = 51.4% / plasma-VAF = 20.5%)

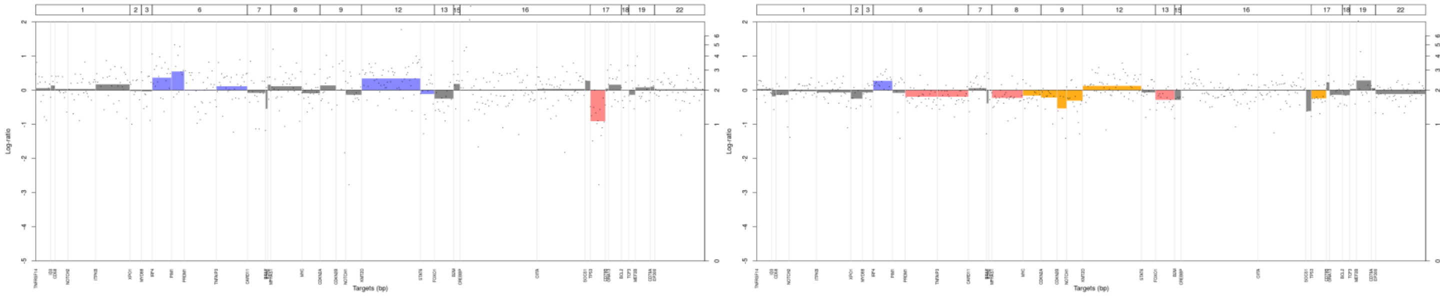

Patient #11 (tumor-VAF = 45.7% / plasma-VAF = 2.5%)

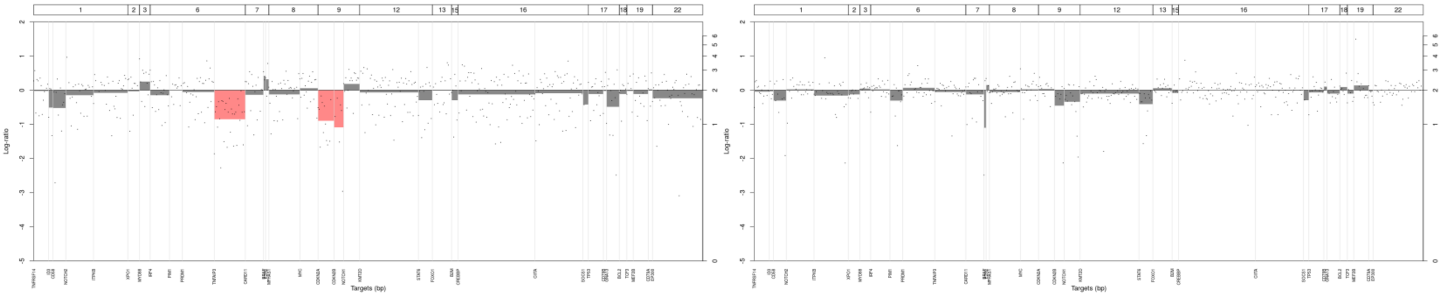

Patient #12 (tumor-VAF = 15.1% / plasma-VAF = 0.04%)

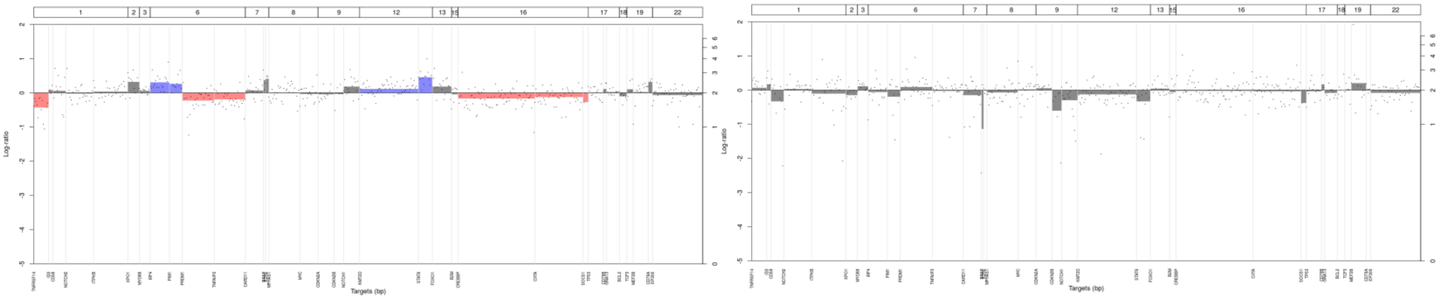

Patient #13 (tumor-VAF = 26.7% / plasma-VAF = 0.49%)

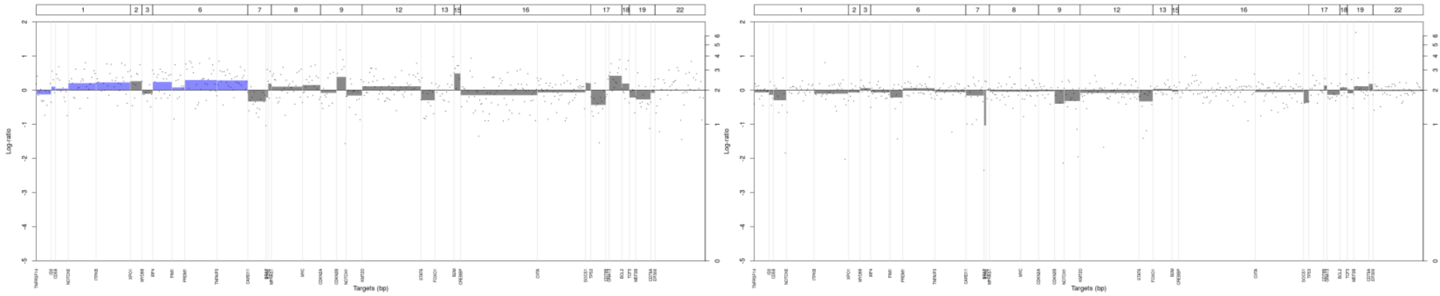

Patient #14 (tumor-VAF = 27.7% / plasma-VAF = 4%)

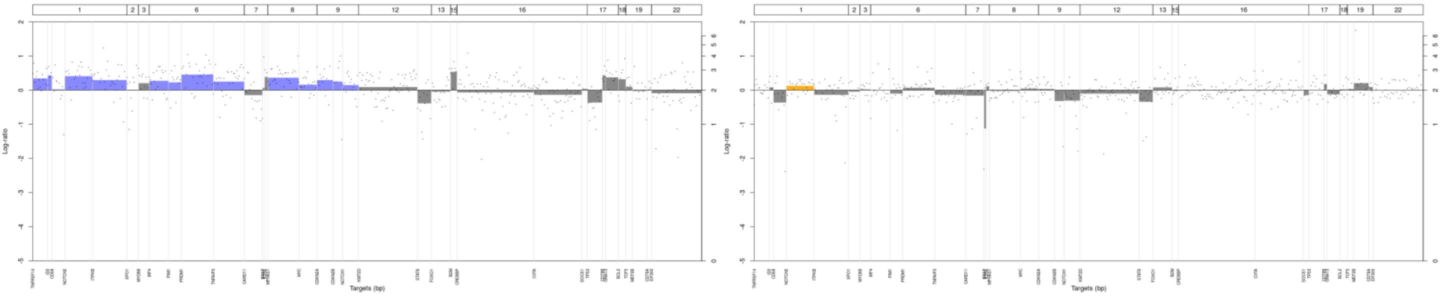

Patient #15 (tumor-VAF = 38.5% / plasma-VAF = 0.04%)

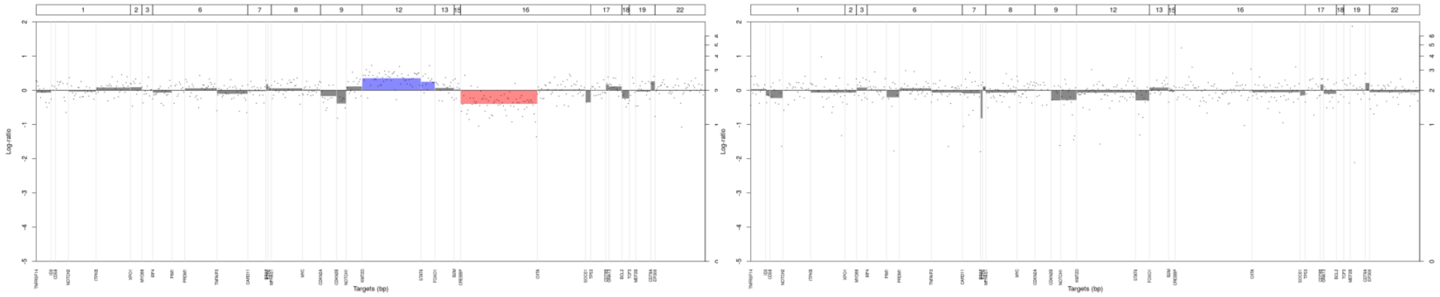

Patient #16 (tumor-VAF = 44.2% / plasma-VAF = 29.8%)

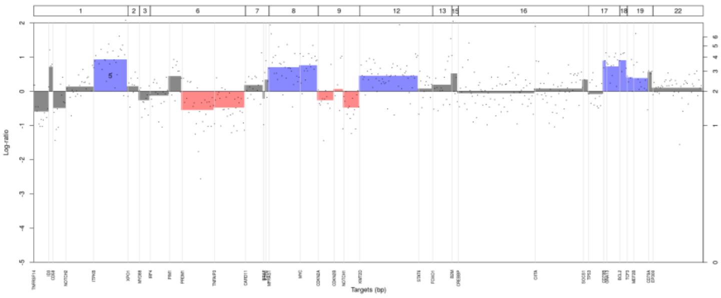

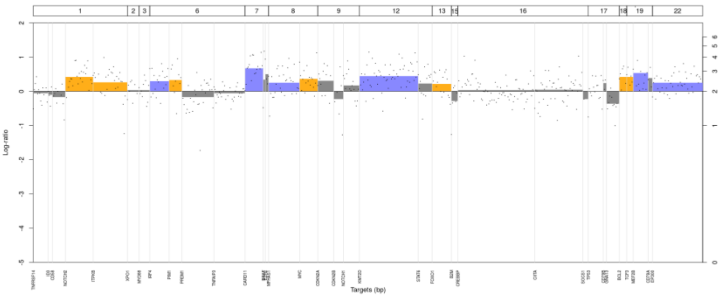

Patient #17 (tumor-VAF = 12% / plasma-VAF = 31.7%)

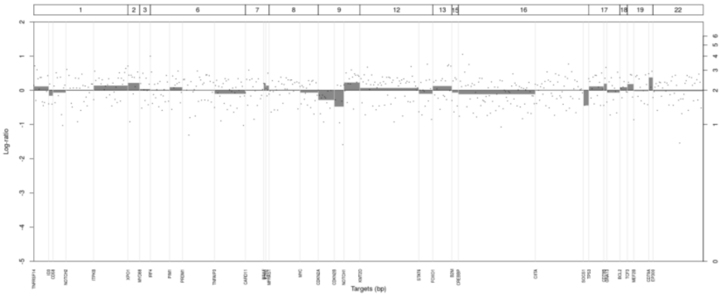

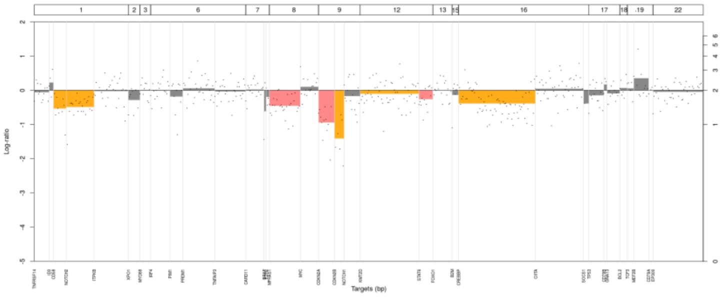

Patient #18 (tumor-VAF = 46.6% / plasma-VAF = 7.6%)

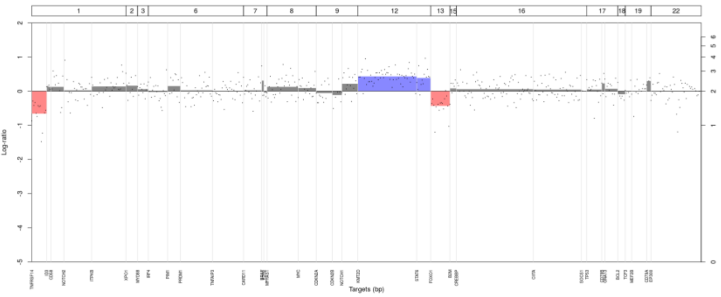

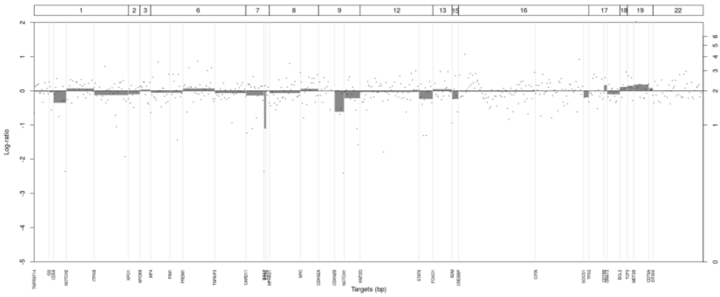

Patient #19 (tumor-VAF = 43.4% / plasma-VAF = 14%)

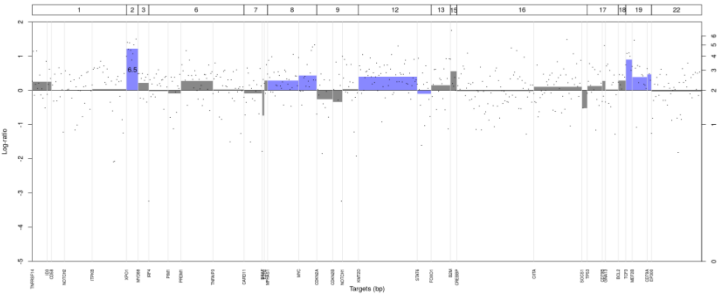

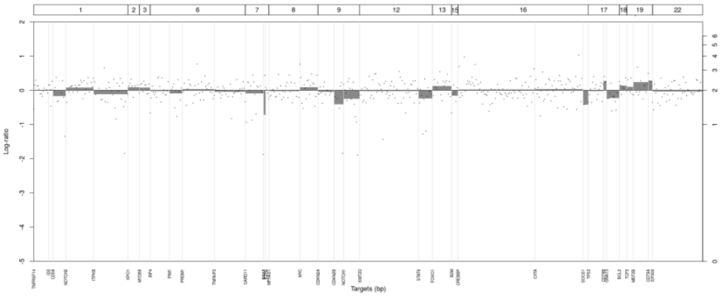

Patient #20 (plasma-VAF = 16.7%)

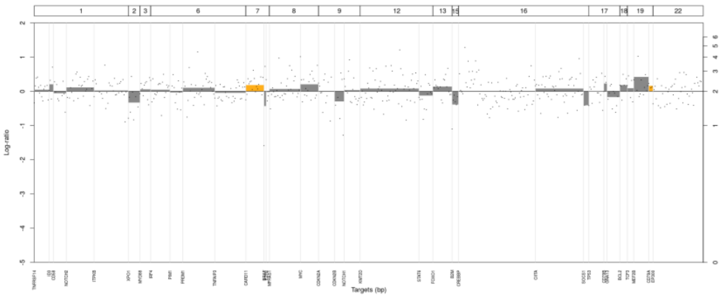

Patient #21 (plasma-VAF = 0%)

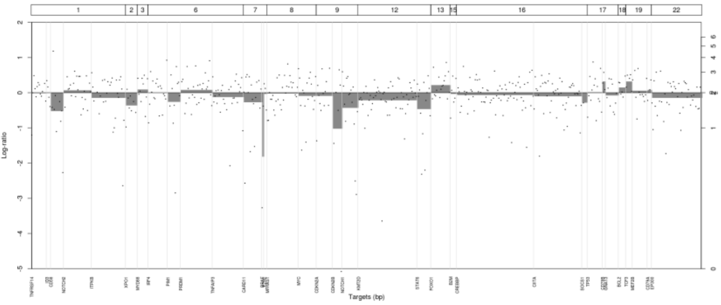

Patient #22 (tumor-VAF = 22.5% / plasma-VAF = 0.39%)

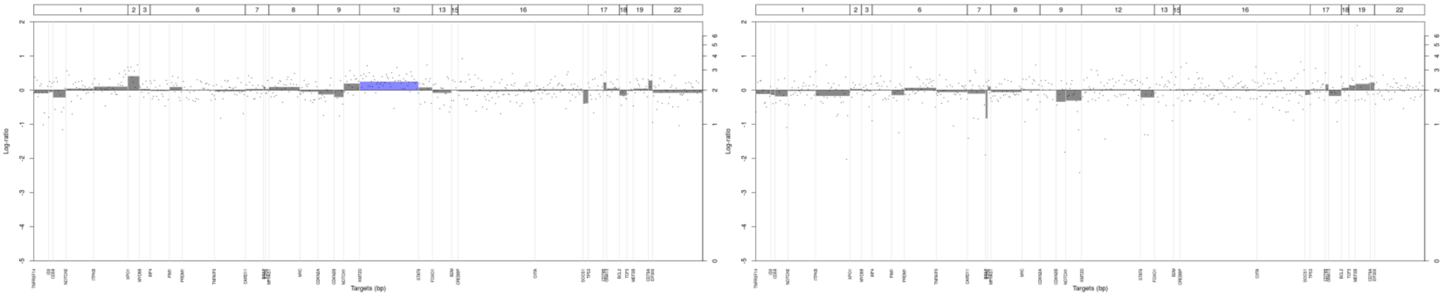

Patient #23 (tumor-VAF = 71.2% / plasma-VAF = 0.65%)

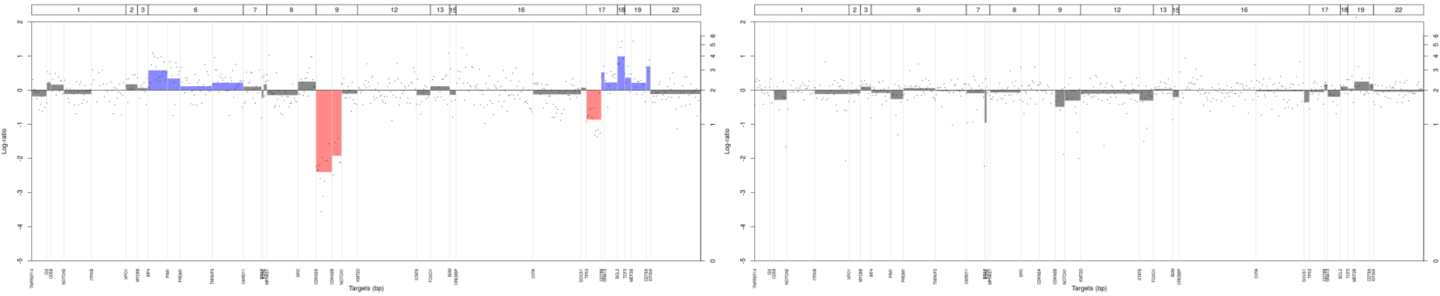

Patient #24 (tumor-VAF = 36.4% / plasma-VAF = 2.8%)

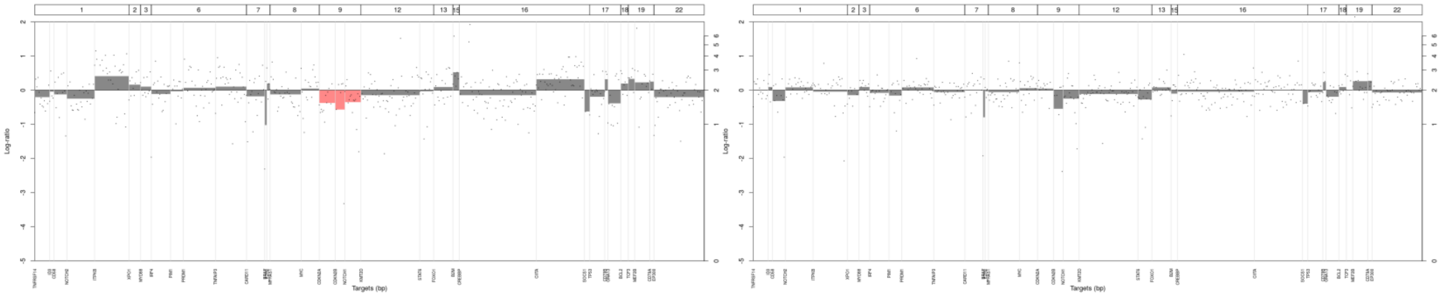

Patient #25 (tumor-VAF = 70.5% / plasma-VAF = 4.6%)

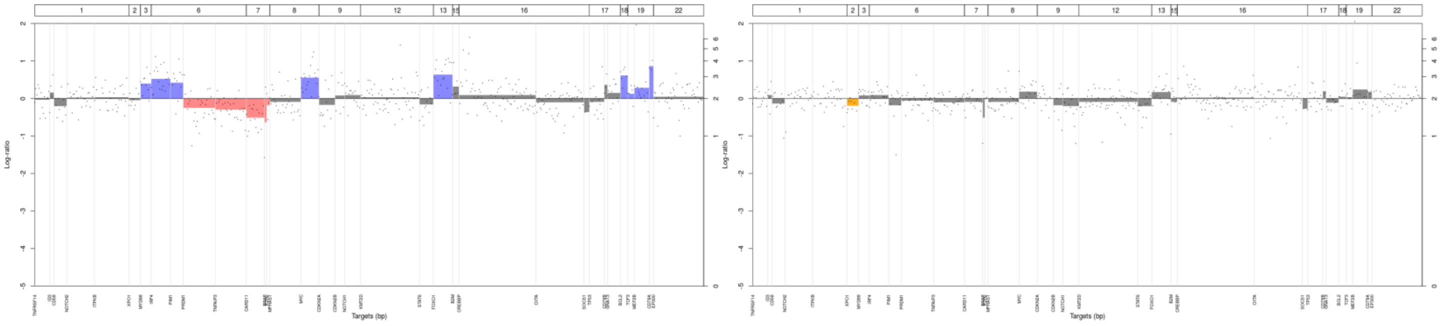

Patient #26 (plasma-VAF = 0%)

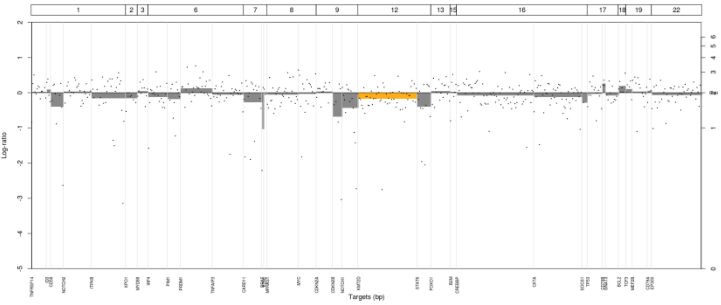

Patient #27 (plasma-VAF = 5.6%)

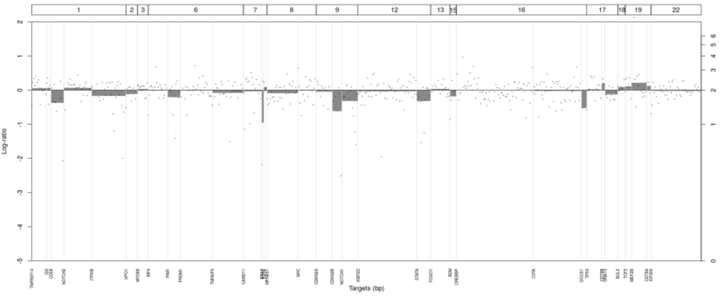

Patient #28 (plasma-VAF = 6.2%)

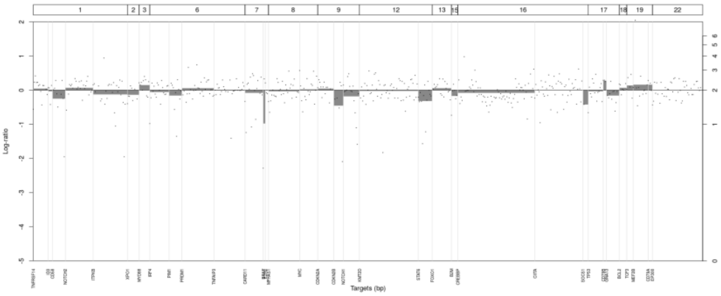

Patient #29 (plasma-VAF = 13.4%)

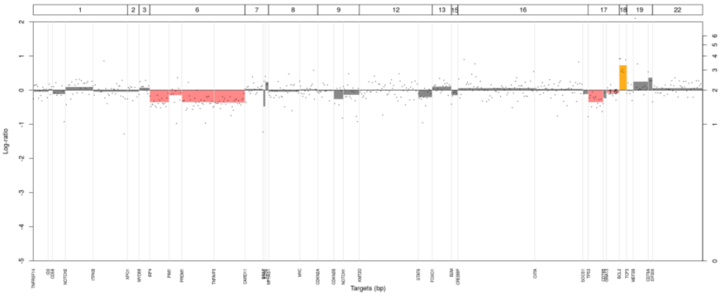

Patient #30 (tumor-VAF = 26.7% / plasma-VAF = 25.5%)

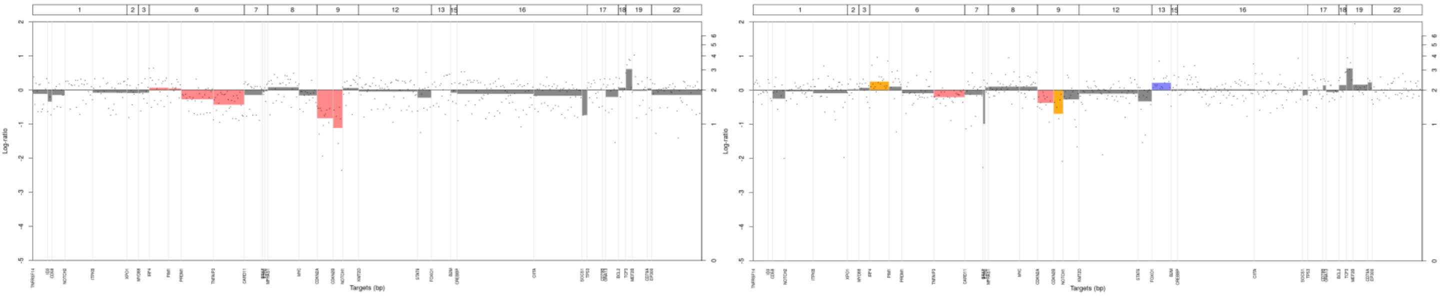

Figure S4: CNV analyses of the 25 tumor DNA and 30 cfDNA available at baseline.

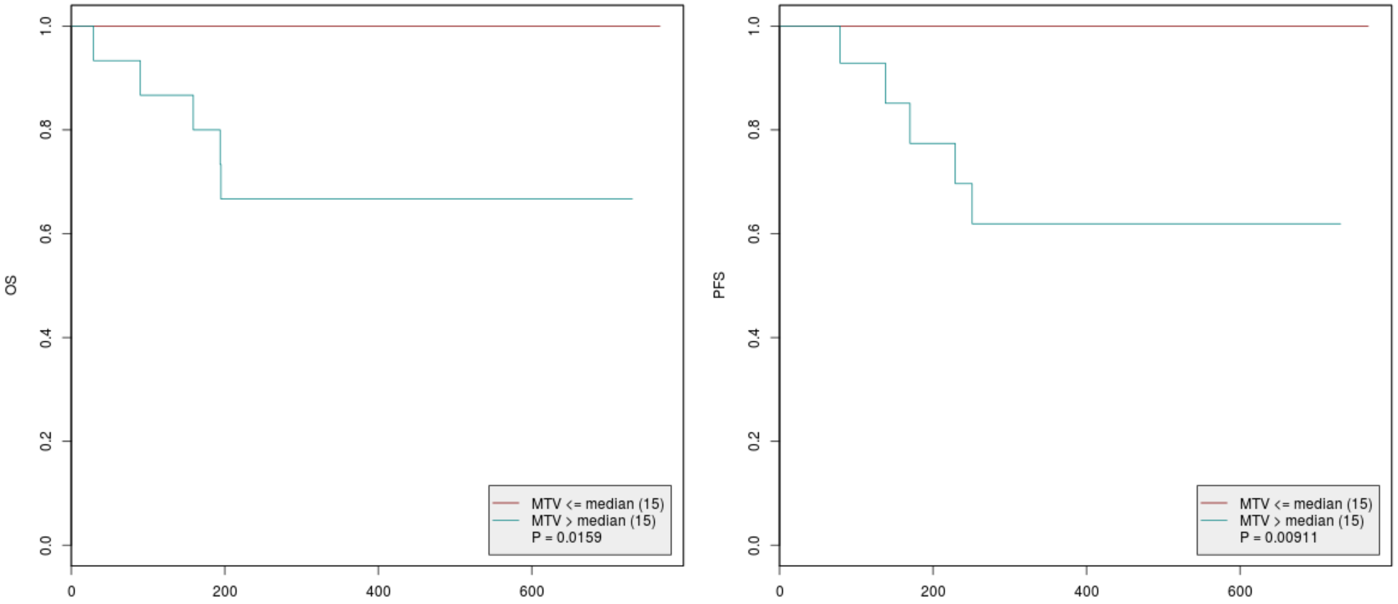

Figure S5: Kaplan-Meier survival curves.
A. Overall and progression-free survival of patients according to the MTV above and below the median (median = 485.5cm^3).
