# Supplementary material for: Non-invasive monitoring of diffuse large B-cell lymphoma by cell-free DNA high-throughput targeted sequencing: analysis of a prospective cohort
Source: Blood Cancer J. 2018 Aug 1;8(8):74. doi: 10.1038/s41408-018-0111-6 (PMC6070497; doi:10.1038/s41408-018-0111-6)
Supplement: Supplementary file 2 — Supplementary Table S1: Mutations and VAF in tumors and plasmas at diagnosis and during follow-up [file 41408_2018_111_MOESM2_ESM.pdf]

patient	Mutations			plasma diagnosis - entire panel			tumour diagnosis			plasma diagnosis -targeted		plasma mid treatment -targe		plasma end treatment -targe		plasma post treatment -targe	
	gene	genomic coordinates	Transcripts	VAF	Number of m	Number of to	VAF	tated reads	total reads	mutated/total	VAF	mutated/total re	VAF	mutated/total r	VAF	mutated/total r	VAF
#1	*PIM1*	chr6:g.37138808G>C	c.240+1G>C	neg			6.77	17	251	0/3714	0.00	0/14623	0	0/33976	0	1/7243	0.0138
	PIM1	chr6:g.37139210C>T	p.L184F				20.98	30	143	7/7165	0.09	135/91844	0.15	33/59641	0.06	13/19123	0.068
	PIM1	chr6:g.37138946G>A	p.V96M				39.66	23	58	2/2156	0.09	2/3484	0.05	2/2052	0.1	0/314	0
	PIM1	chr6:g.37138950G>A	p.S97N				37.93	22	58	0/955	0.00	3/3490	0.09	1/2054	0.05	0/317	0
	PIM1	chr6:g.37138908G>A	p.G83D				40.43	38	94	0/955	0.00	19/10888	0.17	57/25598	0.22	8/4453	0.18
	PIM1	chr6:g.37139063G>A	p.E135K				19.73	88	446	1/7747	0.01	15/71890	0.02	25/122915	0.02	7/27542	0.025
	KMT2D	chr12:g.49433005C>T	p.R2789Q				35.55	75	211	2/2549	0.07	6/5441	0.11	20/20778	0.1	8/7370	0.109
	SOCS1	chr16:g.11348951G>A	p.H129Y				30.75	131	426	5/2988	0.17	8/5772	0.14	13/10611	0.12	8/7846	0.102
	EP300	chr22:g.41566457A>G	p.D1445G				42.83	275	642	7/2236	0.30	4/4083	0.1	15/13150	0.11	6/3627	0.165
	MYD88	chr3:g.38182641T>C	p.L265P				62.48	368	589	40/14690	0.27	143/93593	0.15	307/216162	0.14	101/56340	0.179
	ITPKB	chr1:g.226924388C>G	p.E258Q	neg			12.73	34	267	3/40139	0.007	3/143758	0.002	5/205309	0.002	3/57166	0.005
	MYD88	chr3:g.38182259T>C	p.M232T				21.69	90	415	3/425	0.700	0/176	0	0/521	0	0/121	0
#2	*CARD11*	chr7:g.2977602T>C	p.Y361C				15.76	49	311	140/34178	0.410	218/122240	0.18	394/196407	0.2	85/58845	0.144
	CD79B	chr17:g.62006798T>G	p.Y196S				19.91	43	216	1/19282	0.005	0/10012	0	1/26281	0.004	1/12927	0.008
	CD79A	chr19:g.42384962A>T	p.Y199F				9.7	42	433	4/11260	0.036	0/899	0	0/4001	0	0/1556	0
	SOCS1	chr16:g.11349146A>T	p.Y64N	16.0	159	996	15.91	21	132	275/2265	12.1	1/42655	0.002	3/40814	0	1/90238	0.001
	MFHAS1	chr8:g.8748023T>C	p.K849R	28.4	943	3315	13.99	48	343	564/2444	23.1	40/16844	0.237	35/21614	0.16	44/55175	0.079
#3	*STAT6*	chr12:g.57496077T>C	p.N503S	26.4	523	1984	21.47	38	177	321/1462	22.0	2/2098	0.095	1/1678	0.06	22/14417	0.15
	MFHAS1	chr8:g.8748113A>C	p.L819R	23.9	906	3785	14.72	53	360	10/54	18.5	-	-	-		-	-
	TNFAIP3	chr6:g.138195980A>G	c.296-2A>G	23.9	943	3943	15	72	480	3918/17261	22.7	126/59113	0.43	102/51608	0.2	227/152468	0.15
	MFHAS1	chr8:g.8747786C>G	p.S928T	16.0	529	3310	14.56	82	563	351/2282	15.4	1/52576	0.002	0/50847	0	0/140997	0
	SOCS1	chr16:g.11348971C>T	p.G122E	24.0	974	4066	21.75	77	354	270/998	27.1	0/1524	0	1/1573	0.06	3/17196	0.017
	MFHAS1	chr8:g.8747859T>G	p.S904R	17.4	256	1471	33.33	3	9	89/16593	0.5	-		-	-	-	-
	MFHAS1	chr8:g.8747924A>G	p.I882T	17.3	256	1480	33.33	3	9	92/116	79.3	-		-	-	-	-
	SOCS1	chr16:g.11348988G>C	p.S116R	3.6	213	5927	sanger neg	-	-	0/34	0	0/19	0	0/43	0	0/12	0
	SOCS1	chr16:g.11349004C>G	p.C111S	23.5	440	1871	sanger neg	-	-	10/33	30.3	0/19	0	0/43	0	0/12	0
	SOCS1	chr16:g.11349287C>T	p.A17T	4.6	30	656	sanger faile	-	-	3/105	2.86	1/221	0.45	0/282	0	0/150	0
	SOCS1	chr16:g.11349318C>G	p.Q6H	21.3	140	658	sanger faile	-	-	11/107	10.28	0/220	0	0/284	0	0/150	0
	GNA13	chr17:g.63010835C>A	p.G225V	19.5	417	2142	sanger neg	-	-	6579/33920	19.40	16/22294	0.072	2/35734	0.006	0/33213	0
	GNA13	chr17:g.63052633G>A	p.Q27X	17.9	19	106	sanger pos	-	-	1979/14723	13.44	10/12923	0.077	19/17973	0.106	16/19632	0.081
	TNFAIP3	chr6:g.138202427T>G	p.C782G	14.4	202	1406	sanger neg	-	-	1517/9205	16.48	0/8128	0	1/15598	0.0064	0/7600	0
	MYD88	chr3:g.38182032C>G	p.S219C	14.8	675	4548	26.28	41	156	10081/62890	16.0	55/113939	0.05	NA		NA	
#4	*TNFAIP3*	chr6:g.138197199->GC	p.L234fs	22.8	689	3020	23.58	29	123	1375/6996	19.7	25/6312	0.4				
	TP53	chr17:g.7578538T>A	p.N131I	60.1	1001	1666	70.83	51	72	1501/3812	39.4	13/1071	1.2				
	GNA13	chr17:g.63014404A>C	p.Y176X	25.5	95	373	62.34	48	77	4284/15083	28.4	0/18829	0				
	CD58	chr1:g.117078661->T	p.I185fs	42.23	2047	4847	34.74	132	380	1066/10240	10.4	716/21090	3.3	202/14292	1.4	2980/8501	35
#5	*MYD88*	chr3:g.38182641T>C	p.L265P	51.83	3431	6620	57.35	78	136	12067/23573	51.2	891/31605	2.8	14/18520	0.08	15/10326	0.145
	PIM1	chr6:g.37138765C>G	p.I66M	8.33	344	4131	13.3	29	218	50/914	5.47	0/1222	0	0/1335	0	0/1301	0
	PIM1	chr6:g.37138804G>C	p.E79D	27.48	1243	4524	14.02	15	107	10357/25480	40.65	343/34561	0.99	8/34520	0.023	5/34731	0.014
	PIM1	chr6:g.37138901C>A	p.P81T	39.94	1100	2754	25.4	16	63	9640/23963	29.85	287/32290	0.89	1/32524	0.003	8/31759	0.025
	PIM1	chr6:g.37138950G>C	p.S97T	87.43	292	334	62.9	39	62	342/2209	15.5	87/5394	1.6	0/2498	0	0/335 (?)	0
	PRDM1	chr6:g.106534453C>T	p.R9C	80.83	527	652	60	12	20	1349/1750	77.1	60/8687	0.9	3/7491	0.04	4/2922	0.137
	TNFAIP3	chr6:g.138196122TC>-	p.146_146del	81.50	1159	1422	55.83	115	206	5304/6662	79.6	384/23180	1.7	0/12361	0	0/6190	0
	MYC	chr8:g.128750677C>A	p.P72T	*0.82*	*27*	*3290*	34.04	16	47	29/3150	0.9	1/7690	0.013	4/4310	0.09	3/5658	0.053
	CREBBP	chr16:g.3795277C>A	c.68+1C>A	41.85	1045	2497	28.92	24	83	3595/8002	44.9	460/18467	2.5	2/12614	0.015	0/5754	0
	SOCS1	chr16:g.11348753G>A	p.P195S	52.05	470	903	40.54	15	37	184/351	52.4	25/1409	1.8	4/1362	0.29	3/1541	0.195
	SOCS1	chr16:g.11348897G>A	p.L147F	47.57	2107	4429	37.5	78	208	3684/7457	49.4	186/16819	1.1	5/8820	0.057	2/6610	0.03

	SOCS1	chr16:g.11349286G>C	p.A17G	23.25	199	856	33.33	26	78	129/376	34.3	13/1464	0.88	0/1129	0	0/4304	0
	CD79B	chr17:g.62006799A>T	p.Y196N	44.26	1214	2743	33.77	26	77	4109/9280	42.3	379/22234	1.7	4/12218	0.03	0/5118	0
	EP300	chr22:g.41560079G>A	p.G1251R	47.42	856	1805	32.08	17	53	1899/4312	44.0	84/4389	1.9	0/1532	0	6/5616	0.107
	PIM1	chr6:g.37138630G>A	p.G55D	33.00	528	1600	40	4	10	369/1275	28.94	19/1156	1.64	1/1465	0.068	1/867	0.115
	PIM1	chr6:g.37138600G>A	p.G45D	33.10	521	1574	40	4	10	361/1249	28.90	18/1153	1.56	2/1462	0.137	0/866	0
	ITPKB	chr1:g.226925148G>C	p.Y4X	40.91	9	22	sanger pos	-	-	0	0	0	0	0	0	0	0
	PRDM1	chr6:g.106534460G>A	p.G11D	37.31	244	654	sanger neg	-	-	1241/3868	32.08	15/5301	0.28	1/5259	0.019	3/3004	0.0999
	PIM1	chr6:g.37138308G>A	p.S77N	67.90	55	81	sanger neg	-	-	19/21	90.48	0/30	0	0/7	0	0/5	0
#6	KMT2D	chr12:g.49422962AG>-	p.4711_4711del	neg			32.34	324	1002	0/16	0	0/203	0.000	NA		0/302	0
	KMT2D	chr12:g.49427294G>A	p.Q3732X	1.1	46	4207	8.71	37	425	2/17	11.8	-	-	0	0	0/19	0
#7	BCL2	chr18:g.60985835T>-	p.K22fs	*0.1*	*9*	*7191*	94.5	275	291	2/30	6.7	0	0	0/23	0	0/230	0
	BCL2	chr18:g.60985806C>T	p.A32T	1.1	81	7211	5.48	16	292	0/28	0.0	0	0	0/19	0	0/228	0
	MEF2B,MEF2E	chr19:g.19260064C>T	p.E77K	1.9	111	5878	4.6	16	348	1008/30585	3.3	1/5765	0.017	41/115109	0.04	276/167400	0.16
	BCL2	chr18:g.60985877C>G	p.G8A	neg			11.42	25	219	0/2301	0	0/10378	0	2/43923	0.005	1/8556	0.012
#8	BCL2	chr18:g.60985874T>A	p.Y9F				14.93	33	221	2/2301	0.087	1/10378	0.01	16/43913	0.036	4/8555	0.047
	BCL2	chr18:g.60985592T>C	p.D103G				22.03	52	236	0	0.00	11/12698	0.087	31/21120	0.147	9/4361	0.206
	BCL2	chr18:g.60985508G>T	p.A131D				20.39	95	466	0	0.00	0/12659	0	3/21409	0.014	0/4413	0
	BCL2	chr18:g.60985546CT>GA	pQ118L				40.86	190	465	0	0.00	9/12892	0.07	7/21281	0.033	2/4420	0.045
	EP300	chr22:g.41573120C>T	p.P1802L				61.88	138	223	12514/24647	50.77	9066/18087	50.12	15266/30310	50.3	2767/5455	50.7
	EZH2	chr7:g.148508727T>G	p.Y646S				16.51	224	1357	0/24331	0.00	0/24522	0	1/23749	0.004	0/8096	0
	FOXO1	chr13:g.41240286A>G	p.S22P				25	5	20	6/2340	0.26	2/3212	0.062	8/9133	0.088	0/1289	0
	FOXO1	chr13:g.41239864G>C	p.N162K				12.76	31	243	17/5861	0.29	0/7052	0	1/15403	0.006	0/2615	0
	FOXO1	chr13:g.41239872A>T	p.W160R				12.92	31	240	23/5829	0.39	1/6963	0.014	0/15207	0	0/2580	0
	GNA13	chr17:g.63052610G>C	p.I34M				41.67	5	12	9/3923	0.23	0/5230	0	0/4554	0	0/1513	0
	KMT2D	chr12:g.49446010A>G	p.S486P				7.14	8	112	67/33253	0.20	56/25500	0.22	75/47279	0.159	15/9751	0.154
	MEF2B,MEF2E	chr19:g.19260088A>G	p.Y69H				32.31	63	195	440/72711	0.61	44/51258	0.086	67/59146	0.113	24/12343	0.194
	PIM1	chr6:g.37138615G>A	p.G50D				41.67	10	24	18/2845	0.63	0/1181	0	0/2638	0	1/474	0.211
	PIM1	chr6:g.37139063G>T	p.E135X				35.4	57	161	55/19888	0.28	0/14432	0	0/22177	0	0/6933	0
	STAT6	chr12:g.57496658T>G	p.N420T				26.44	147	556	1/20173	0.00	2/11002	0.018	3/18611	0.016	0/3684	0
	TNFRSF14	chr1:g.2489898T>G	p.C99G				12.4	16	129	4/3713	0.11	2/6156	0.032	2/27218	0.007	1/3729	0.027
	TNFRSF14	chr1:g.2494611G>T	p.E251X				28.71	60	209	342/52861	0.65	1/37559	0.002	1/66848	0.002	1/11231	0.009
#9	STAT6	chr12:g.57499078T>C	p.Q286R	12.06	393	3258	14.7	61	415	1101/9469	11.63	25/11497	0.217	42/19044	0.221	65/17844	0.364
	B2M	chr15:g.45003781CT>-	p.13_13del	21.38	257	1202	27.86	56	201	3244/14488	22.39	12/18935	0.063	256/28460	0.8995	1/27982	0.0036
	CARD11	chr7:g.2979499A>G	p.S250P	24.53	589	2401	44	33	75	816/3039	26.85	3/2646	0.11	NA		NA	
#10	CARD11	chr7:g.2985458A>G	p.I118T	25.07	856	3415	42.62	26	61	6107/24941	24.49	13/18173	0.07			(seq moelle neg)	
	KMT2D	chr12:g.49427489G>A	p.Q3667X	31.29	924	2953	57.89	11	19	0/1	0	0	0				
	B2M	chr15:g.45003745A>T	p.M1L	28.42	418	1471	50.51	50	99	1904/6845	27.82	1/29634	0				
	B2M	chr15:g.45003781CT>-	p.13_13del	14.57	213	1462	40.4	40	99	935/6057	15.44	8/29757	0.03				
	CREBBP	chr16:g.3781324AGG>-	p.1680_1681del	14.33	508	3544	33.33	6	18	1960/13520	14.5	21/12576	0.17				
	TP53	chr17:g.7577538C>T	p.R248Q	*0.001*	*6*	*5564*	93.9	77	82	85/75196	0.11	80/93284	0.09				
	GNA13	chr17:g.63052543C>G	p.G57R	11.51	232	2015	41.51	22	53	2061/17168	12	0/22253	0				
	MEF2B,MEF2E	chr19:g.19260045T>A	p.D83V	17.60	519	2949	58.62	17	29	8931/54847	16.28	1/37863	0				
	CIITA	chr16:g.11016349T>G	c.3317+2T>G	6.57	570	8682	*0*	*0*	*144*	4/98	4.08	0/226	0				
	TP53	chr17:g.7577517A>G	p.I255T	36.92	1985	5377	*0*	*0*	*80*	9683/25926	37.35	101/47628	0.212				
	MYD88	chr3:g.38182641T>C	p.L265P	1.89	98	5184	30	99	330	210/12636	1.66	13/11019	0.11	15/17966	0.08	76/70119	0.11
#11	PIM1	chr6:g.37138765C>G	p.I66M	*0.003*	*10*	*3262*	49.66	74	149	1/370	0.27	0/159	0	0/6398	0	0/3224	0
	CD79B	chr17:g.62006799A>T	p.Y196N	3.77	108	2864	74.39	122	164	49/3051	1.60	0/826	0	0/1396	0	2/8348	0.024
	KMT2D	chr12:g.49416416C>T	p.R5432Q	1.87	53	2832	28.92	24	83	14/694	2.02	0/451	0	1/904	0.11	4/6800	0.059
	PIM1	chr6:g.37138905A>G	p.N82S	neg			9.26	5	54	1/7983	0.013	0/26	0	4/14274	0.028	2/13308	0.015

#12	*SOCS1*	chr16:g.11348972C>T	p.G122R				19.65	67	341	0/1280	0.000	0/2	0	2/5497	0.036	0/3731	0
	TP53	chr17:g.7576870C>A	p.E326X				16.4	51	311	2/31677	0.006	0/4558	0	2/62086	0.003	0/31331	0
	MYD88	chr3:g.38182032C>G	p.S219C	*0.54*	*29*	*5331*	20.52	55	268	412/56425	0.73	35/161902	0.02	35/188426	0.02	36/236261	0.015
#13	*PIM1*	chr6:g.37139061C>T	p.T134M	*0.58*	*23*	*3934*	24.87	47	189	71/16252	0.44	18/47233	0.04	29/69047	0.04	27/74623	0.036
	PIM1	chr6:g.37139210C>T	p.L184F	*0.55*	*3*	*543*	32.79	20	61	12/1577	0.76	10/11076	0.09	8/7058	0.11	18/33424	0.054
	KMT2D	chr12:g.49420213C>T	p.R5179H	*0.68*	*40*	*5856*	17.96	65	362	274/32713	0.84	42/89889	0.05	174/139909	0.12	210/112543	0.19
	FOXO1	chr13:g.41239755C>T	p.D199N	*0.47*	*27*	*5780*	37.5	60	160	0/41	0.00	0/3	0	0/78	0	0/304	0
	SOCS1	chr16:g.11348810CCTGCA	p.169_176del	2.2	87	3886	25.13	50	199	8/395	2.0	0	0	NA		0/5730 (?)	0
#14	*SOCS1*	chr16:g.11348906AGCTCT	p.139_144del	3.3	187	5673	38.22	120	314	106/3220	3.3	-	-			0/31030 (?)	0
	CD58	chr1:g.117078654A>T	p.C187X	3.28	65	1984	25.88	198	765	857/13136	6.5	2/79270	0			1/82527	0.0012
	ITPKB	chr1:g.226924583G>A	p.Q193X	2.5	66	2612	28.57	14	49	11/45	24.4	0/49	0			0/936	0
	ITPKB	chr1:g.226924673C>A	p.A163S	2.2	175	8019	33.81	71	210	243/3697	6.6	0/1492	0			0/46333	0
	MFHAS1	chr8:g.8749759G>C	p.S270R	4.15	176	4237	19.66	35	178	629/14955	4.2	0/43727	0			2/88488	0.002
	CIITA	chr16:g.10971239G>C	p.G18R	6.05	251	4147	29.41	45	153	1677/21519	7.8	0/45621	0			0/67853	0
	SOCS1	chr16:g.11348867C>G	p.A157P	-	-	-	13.61	26	191	0/293	0.0	0	0			0/5295	0
	SOCS1	chr16:g.11348875T>A	p.Y154F	5.92	178	3008	31.41	49	156	13/528	2.5	0	0			2/5083	0.039
	SOCS1	chr16:g.11348878T>C	p.H153R	5.99	179	2986	31.82	49	154	13/246	5.3	0	0			2/4855	0.041
	GNA13	chr17:g.63010482A>T	p.Y343N	5.61	101	1799	26.89	82	305	303/5553	5.5	0/28502	0			0/21982	0
	BRAF	chr7:g.140453153A>C	p.D594E	neg			31.97	164	513	0/1677	0	0/3801	0	0/2665	0	0/1143	0
#15	*EZH2*	chr7:g.148508728A>G	p.Y646H				37.1	174	469	5/36860	0.0136	9/35910	0.0251	5/26912	0.0186	5/19535	0.0256
	FOXO1	chr13:g.41240279G>A	p.T24I				46.3	25	54	6/18558	0.0323	16/19770	0.0809	11/15258	0.0721	4/7554	0.0530
							difficult to interpret										
	KMT2D	chr12:g.49426961G>A	p.Q3843X	37.91	2155	5685	49.32	253	513	-		NA		NA		NA	
#16	*KMT2D*	chr12:g.49434634C>T	p.V2307I	*0*	*0*	*162*	12.12	8	66								
	KMT2D	chr12:g.49441781A>-	p.S1401fs	35.09	2596	7398	41.03	382	931								
	GNA13	chr17:g.63052492G>A	p.Q74X	3.65	45	1234	*0*	*0*	*373*								
	TP53	chr17:g.7578454G>A	p.A159V	56.46	883	1564	91.98	195	212								
	BCL2	chr18:g.60985361T>A	p.Y180F	14.92	413	2769	30.29	159	525								
	BCL2	chr18:g.60985536T>C	p.T122A	16.05	2174	13544	34.1	581	1704								
	BCL2	chr18:g.60985883C>T	p.R6K	11.74	419	3570	28.75	140	487								
	PRDM1	chr6:g.106553383C>T	p.L450F	22.69	444	1957	84.65	171	202								
	EZH2	chr7:g.148508727T>G	p.Y646S	17.30	710	4103	25.15	86	342								
	MYC	chr8:g.128748858G>A	p.V7M	14.16	791	5587	*0.08*	*1*	*1236*								
	B2M	chr15:g.45003781CT>-	p.13_13del	22.85	61	267	16.22	30	185	58/16416	0.353	2/24685	0.008	10/25018	0.040	1/21624	0.0046
#17	*KMT2D*	chr12:g.49445464C>A	p.E668X	31.57	1617	5122	*14.29*	*10*	*70*	574/1781	32.23	0/1582	0	0/1144	0	1/1591	0.063
	FOXO1	chr13:g.41240273G>A	p.P26L	30.94	298	963	*5.56*	*2*	*36*	2747/8371	32.82	10/10400	0.096	8/15363	0.052	11/15062	0.073
							strand bias filtered										
	EZH2	chr7:g.148508727T>A	p.Y646F	6.29	223	3545	35.47	155	437	2541/40717	6.24	0/37532	0	2/34872	0.0057	0/34636	0
#18	*KMT2D*	chr12:g.49415846G>A	p.R5501X	14.12	440	3117	62.62	268	428	2/24	8.33	0/39	0	0/40	0	0/27	0
	CREBBP	chr16:g.3789578C>G	c.77+1C>G	6.93	173	2497	44.5	89	200	103/1999	5.15	0/2555	0	0/3363	0	0/2333	0
	EP300	chr22:g.41554459T>A	p.L1182X	5.16	19	368	39.13	9	23	246/4742	5.19	0/3955	0	1/4777	0.021	0/3736	0
	BCL2	chr18:g.60985803C>T	p.G33R	5.65	391	6915	51.28	60	117	421/13519	3.11	13/20269	0.064	6/26514	0.0226	10/19625	0.0510
							difficult to interpret										
#19	*TP53*	chr17:g.7579313G>T	p.T125K	13.95	275	1971	43.39	174	401	781/4361	17.91	349/8069	4.3251952	329/6466	5.0881534	481/4509	10.6675538
	BCL2	chr18:g.60985311C>T	p.G197S	6.99	69	987	NA			195/2885	6.8	NA		0/1999	0	3/5427	0.055
#20	*TNFRSF14*	chr1:g.2488105T>C	p.M1T	11.13	62	557				484/3700	13.1			4/3810	0.105	11/6671	0.165
	EZH2	chr7:g.148508727T>G	p.Y646S	13.75	131	953				325/2338	13.9			0/1774	0	0/4984	0
	SOCS1	chr16:g.11348864G>T	p.P158T	13.33	173	1298				571/4579	12.5			0/3983	0	0/9350	0
	MYC	chr8:g.128750945C>T	p.S161L	15.56	164	1054				220/1195	18.4			0/511	0	0/2767	0

	CARD11	chr7:g.2979557ATC>-	p.230_230del	12.72	234	1839				693/4345	15.9			0/3081	0	0/7182	0
	CREBBP	chr16:g.3790523T>G	p.H1337P	15.4	248	1610				1747/12974	13.5			0/6741	0	1/11083	0.009
	TNFRSF14	chr1:g.2489821C>T	p.T73I	15.74	751	4771				3776/22626	16.7			6/9573	0.063	16/20757	0.077
	KMT2D	chr12:g.49433015C>-	p.V2786X	19.84	253	1275				400/1763	22.7			0/2159	0	0/5187	0
	KMT2D	chr12:g.49441849TGTCC>	p.1378_1379del	21.46	426	1985				1352/4030	33.5			2/2554	0.078	5/4766	0.105
#21	*neg*			neg			NA										
	STAT6	chr12:g.57499078T>C	p.Q286R				26.92	91	338	296/28732	1.03	12/7368	0.16	16/7108	0.23	20/8144	0.25
#22	*SOCS1*	chr16:g.11348889C>G	p.E149D	neg			15.38	32	208	0/1	0.00	0/31	0	0/40	0	0/93	0
	SOCS1	chr16:g.11348908C>T	p.S143N				13.74	29	211	0/1	0.00	0/31	0	0/40	0	0/94	0
	SOCS1	chr16:g.11348924C>T	p.D138N				4.78	10	209	0/0	0.00	0/32	0	0/42	0	0/99	0
	SOCS1	chr16:g.11348927GGTGA	p.133_137del				30.92	64	207	0/0	0.00	0/32	0	0/42	0	0/99	0
	SOCS1	chr16:g.11348951G>T	p.H129N				30.66	65	212	0/0	0.00	0/32	0	0/42	0	0/98	0
	SOCS1	chr16:g.11348962C>G	p.S125T				30.33	64	211	0/0	0.00	0/31	0	0/42	0	0/98	0
	SOCS1	chr16:g.11348988G>T	p.S116R				8.68	23	265	0/7	0.00	-	-	-	-	0/102	0
	SOCS1	chr16:g.11349033G>T	p.F101L				22.73	15	66	0/4	0.00	-	-	-	-	-	-
	SOCS1	chr16:g.11349139C>T	p.R66H				20.83	5	24	30/2110	1.42	22/19071	0.12	22/38108	0.06	23/24164	0.095
	SOCS1	chr16:g.11349146A>C	p.Y64D				27.27	6	22	12/2204	0.54	0/19062	0	1/38082	0.003	0/24451	0
	SOCS1	chr16:g.11349160C>T	p.R59H				38.1	8	21	39/2210	1.76	13/18603	0.07	70/37948	0.18	42/25169	0.17
	SOCS1	chr16:g.11349162G>C	p.F58L				27.78	5	18	12/2210	0.54	0/18584	0	0/37927	0	0/25248	0
	SOCS1	chr16:g.11349209G>A	p.P43S				27.59	8	29	36/2235	1.61	5/16216	0.031	11/35567	0.03	19/25425	0.075
	TNFAIP3	chr6:g.138192559C>G	p.H65Q				15.94	33	207	84/21802	0.39	-	-	-	-	-	-
	PIM1	chr6:g.37138775G>A	p.E70K				12.18	24	197	7/8086	0.09	-	-	-	-	-	-
	MFHAS1	chr8:g.8749850C>T	p.G240D				35.25	43	122	46/4795	0.96	8/4649	0.17	14/7352	0.19	11/9976	0.11
	MFHAS1	chr8:g.8750085C>T	p.E162K				15.89	17	107	3/987	0.30	0/14	0	0/18	0	0/24	0
	PIM1	chr6:g.37139210C>T	p.L184F	*0.5602*	*4*	*714*	38.1	48	126	59/3603	1.64	83/12547	0.66	NA		NA	
#23	*TP53*	chr17:g.7577082C>T	p.E286K	*0.7326*	*22*	*3003*	81.91	77	94	29/1397	1.65	12/3544	0.33				
	PRDM1	chr6:g.106536076G>A	p.A15T	neg			82.42	347	421	54/15223	0.35	58/48221	0.12				
	PRDM1	chr6:g.106552768->AG	p.K245fs				82.52	878	1064	325/16840	1.93	153/8294	1.8				
	BCL2	chr18:g.60985555C>G	p.M115I	1.29	79	6143	8.33	20	240	245/46001	0.53	0/5116	0	0/2727	0	1/10502	0.009
#24	*BCL2*	chr18:g.60985575G>A	p.R109C	1.38	85	6145	9.17	22	240	315/46059	0.68	2/5092	0.04	3/2709	0.111	10/10501	0.095
	BCL2	chr18:g.60985802C>T	p.G33E	0.92	69	7470	23.64	52	220	2/2796	0.07	2/3996	0.05	1/8098	0.012	3/13801	0.021
	BCL2	chr18:g.60985433A>G	p.V156A	2.14	75	3508	42.86	111	259	753/42985	1.75	83/28969	0.29	73/38585	0.189	104/42980	0.242
	BCL2	chr18:g.60985342G>C	p.H186Q	1.81	64	3539	44.61	120	269	659/43220	1.52	0/30081	0	0/39209	0	0/45168	0
	BCL2	chr18:g.60985327A>C	p.D191E	1.81	64	3532	44.78	120	268	659/42975	1.53	3/21681	0.014	2/23912	0.008	3/25030	0.012
	CD58	chr1:g.117078661->T	p.I185fs	4.17	198	4750	22.21	241	1085	1465/41238	3.55	-	-	-	-	-	-
	ITPKB	chr1:g.226924345G>A	p.A272V	2.60	178	6847	22.32	323	1447	148/4195	3.53	1/1923	0.05	1/1343	0.074	1/3613	0.028
	SOCS1	chr16:g.11349004C>T	p.C111Y	2.33	71	3052	33.82	23	68	12/27	44.44	0/24	0	0/40	0	0/99	0
	SOCS1	chr16:g.11348798G>T	p.Q180K	6.66	200	3003	51.06	24	47	26/220	11.82	0/47	0	0/24	0	1/562	0.178
	SOCS1	chr16:g.11348812T>A	p.Q175L	6.21	187	3012	52.17	24	46	29/223	13.00	0/47	0	0/24	0	1/557	0.179
	SOCS1	chr16:g.11348796C>G	p.Q180H	6.24	187	2999	53.19	25	47	24/218	11.01	0/47	0	0/23	0	0/562	0
	SOCS1	chr16:g.11348920C>T	p.G139D	2.17	93	4280	42.33	80	189	0/25	0.00	-	-	-	-	-	
	SOCS1	chr16:g.11348908C>G	p.S143T	2.12	91	4287	43.68	83	190	0/25	0.00	-	-	-	-	-	
	SOCS1	chr16:g.11348927G>C	p.L137V	1.89	81	4276	44.21	84	190	0/25	0.00	-	-	-	-	-	
	SOCS1	chr16:g.11348951G>A	p.H129Y	2.21	94	4252	44.44	84	189	0/26	0.00	-	-	-	-	-	
#25	*PRDM1*	chr6:g.106547327->CC	p.K188fs	2.6	79	3024	71.2	220	309	15/194	7.73	1/54	1.85	NA		NA	
	TNFAIP3	chr6:g.138192575->TT	p.R71fs	6.5	108	1657	69.74	318	456	2399/33199	7.23	53/5554	0.95				
							NA										

#26	*neg*			neg													
	PIM1	chr6:g.37139001A>G	p.D114G	4.05	16	395	NA			32/1733	1.8	5/1706	0.29	2/2016	0.0992063	5/1809	0.2763958
#27	*B2M*	chr15:g.45007898G>A	p.W115X	7.27	288	3962				12/181	6.6	0/542	0.00	0/303	0	0/180	0
	TNFRSF14	chr1:g.2488173G>A	c.69+1G>A	8.37	151	1804				2246/27269	8.2	19/19603	0.10	13/18019	0.0721461	22/26376	0.08340916
							sequencing failed										
#28	*PRDM1*	chr6:g.106554273C>T	p.R601W	6.30	259	4108	*0.002*	*2*	*1054*	2135/39031	5.5	14/28634	0.049	31/36531	0.0848594	14/32568	0.04298698
	TP53	chr17:g.7578236A>C	p.Y205D	6.07	322	5301	*0*	*0*	*610*	2/80	2.5	0/218	0	0/96	0	0/133	0
	TP53	chr17:g.7577538C>T	p.R248Q	38.57	1932	5009	NA			4325/11231	38.5	603/14417	4.183	NA		NA	
#29	*PIM1*	chr6:g.37139063G>A	p.E135K	7.56	256	3385				654/8314	7.9	3/7633	0.039				
	PIM1	chr6:g.37139210C>T	p.L184F	8.23	61	741				249/4027	6.2	2/6340	0.032				
	PIM1	chr6:g.37138772G>A	p.V69M	6.1	225	3754				279/6785	4.1	4/7725	0.052				
	FOXO1	chr13:g.41239875C>T	p.A159T	6.5	257	3983				386/7522	5.1	18/6635	0.271				
	IRF4	chr6:g.393222C>A	p.L24I	7.1	213	3006				319/4099	7.8	158/7349	2.150				
	IRF4	chr6:g.394903C>T	p.A100V	9.6	325	3374				518/6170	8.4	263/10055	2.616				
	IRF4	chr6:g.393215C>A	p.N21K	9.8	293	3001				374/4036	9.3	160/7317	2.187				
	BCL2	chr18:g.60985793C>T	p.G36D	12.6	1340	10616				1261/9115	13.8	235/7039	3.339				
	PRDM1	chr6:g.106547174G>A	c.412-1G>A	40.28	669	1661				1364/4182	32.6	205/6016	3.408				
	PIM1	chr6:g.37139210C>T	p.L184F	5.97	40	670	18.31	39	213	220/2078	10.59	NA		0/579	0	3/5046	0.06
#30	*MYD88*	chr3:g.38182641T>C	p.L265P	22.78	1310	5750	22.17	98	442	69/370	18.65			0/686	0	1/1722	0.06
	CD79B	chr17:g.62006798T>C	p.Y196C	47.73	2306	4831	39.66	117	295	18/43	41.86			0/30	0	0/85	0

Supplementary Table S1: Mutations and VAF in tumors and plasmas at diagnosis and during follow-up.
data in italic represents mutations not detected by the variant caller and analysed with IGV software
