# Supplementary material for: Non-invasive monitoring of diffuse large B-cell lymphoma by cell-free DNA high-throughput targeted sequencing: analysis of a prospective cohort
Source: Blood Cancer J. 2018 Aug 1;8(8):74. doi: 10.1038/s41408-018-0111-6 (PMC6070497; doi:10.1038/s41408-018-0111-6)
Supplement: Supplementary file 3 — Supplementary Table S2: Patients’ data [file 41408_2018_111_MOESM3_ESM.pdf]

patients	Diagnosis												[illegible]	
	Age	Sexe	LDH	IPI	Stage	Subtype	VMT	TLG	mean VAF tumor (%)	mean VAF plasma (%)	hGE/mL	SUVmax	VAF mi-ttmt	delta SUV max
#1	78	M	372	3	IV	Non-GCB	64.14	857.00	33.71	0.06	5.74	23.01	0.098	91.31
#2	74	M	256	1	II	Non-GCB	20.19	52.00	15.96	0.13	7.31	17.14	0.036	81.62
#3	25	F	576	2	IV	Und (PMBL)	294.74	3446.00	20.45	18.62	1678.96	43.03	0.128	84.29
#4	93	M	699	2	II	GCB	521.91	5020.00	45.76	30.80	27776.00	22.84	0.413	81.61
#5	53	M	2940	4	IV	Non-GCB	2441.14	24511.00	37.87	44.42	22507.91	30.07	1.536	91.92
#6	59	M	431	0	IIE	GCB	40.17	339.00	32.34	0.00	0.00	15.76	0.000	86.04
#7	59	M	362	1	IV	GCB	100.02	640.00	28.32	1.31	125.21	13.21	0.017	-28.01
#8	59	M	464	2	IV	GCB	713.55	5682.00	25.80	0.54	49.16	19.04	0.035	94.75
#9	20	F	336	0	II	GCB (PMBL)	373.05	4747.00	21.28	16.60	1631.00	21.96	0.000	89.07
#10	73	F	2411	5	IV	GCB	2846.62	20572.00	51.42	20.49	9084.34	26.00	0.080	79.19
#11	72	F	403	3	IV	GCB	40.57	401.00	45.74	2.51	141.15	18.94	0.028	87.86
#12	66	M	304	2	III	GCB	6.4	20.00	15.10	0.04	4.18	5.54	0.000	81.95
#13	65	M	330	1	IIE	Non-GCB	54.8	647.00	26.73	0.49	22.14	23.29	0.050	95.71
#14	34	M	795	0	II	Non-GCB (PMBL)	425.51	5648.00	27.67	4.04	477.35	29.19	0.000	90.13
#15	69	M	299	1	I	GCB	0.29	1.17	38.46	0.04	1.84	4.66	0.008	78.54
#16	68	F	1663	4	IV	GCB	2740.06	31149.00	44.15	29.84	39151.31	22.01	NA	-
#17	64	M	1561	4	IV	GCB	2374.9	31100.00	12.02	31.66	15110.45	33.04	0.000	96.97
#18	73	M	508	3	III	GCB	300.04	1476.00	46.60	7.63	493.67	10.20	0.005	67.94
#19	84	F	404	2	IE	GCB	128.08	787.00	43.39	13.95	1465.60	12.52	4.325	34.90
#20	57	M	562	3	IV	GCB	2015.58	26779.00	NA	16.70	9810.25	24.36	NA	88.10
#21	81	F	446	3	IV	Und	438.82	6040.00	NA	0.00	0.00	25.72	0.000	53.77
#22	35	F	450	2	IV	Non-GCB (PMBL)	464.1	4648.00	22.50	0.39	42.20	27.20	0.079	77.90
#23	78	F	470	3	IV	Non-GCB	622.00	8792.00	71.24	0.65	49.74	25.68	0.353	14.76
#24	75	F	385	3	III	GCB	219.17	2947.00	36.43	2.82	202.05	31.57	0.063	94.62
#25	64	F	2512	5	III	Non-GCB	932.58	11484.00	70.47	4.55	1322.26	23.46	1.400	74.85
#26	79	F	483	4	IV	Und	258.98	2663.59	NA	0.00	0.00	17.68	0.000	21.44
#27	39	M	462	2	IV	GCB	450.39	7736.00	NA	5.57	635.93	31.96	0.130	96.87
#28	77	M	222	4	IV	Non-GCB	1560.87	24144.00	NA	6.19	906.55	32.69	0.024	78.07
#29	84	F	581	5	IV	Und	1571.3	14733.00	NA	13.37	3403.21	19.11	1.827	-11.41
#30	64	M	440	1	I	Non-GCB	290.71	3197.00	26.71	25.49	2000.84	27.51	NA	86.99

Supplementary Table S2: Patients' data.

PR = partial response (residual disease at the end of treatment)
CR = complete response
S = stable (absence of metabolic response)
D = Death
NA = Not available

Mid-treatment			End of treatment			Post treatment / end of trial	
Deauville Score	Response (Deauville)	Response (deltaSUVmax)	VAF fin-ttmt	Deauville Score	Response (Lugano)	VAF post-ttmt	disease status
3	CR	CR	0.092	1	CR	0.084	CR
2	CR	CR	0.041	1	CR	0.031	CR
4	PR	CR	0.080	4	PR	0.066	CR
3	CR	CR	NA	D	-	NA	D
3	CR	CR	0.055	3	CR	0.059	CR
2	CR	CR	NA	1	CR	0.000	CR
5	S	S	0.040	4	PR	0.053	CR
1	CR	CR	0.037	5	progression	0.053	progression
2	CR	CR	0.050	1	CR	0.000	CR
4	PR	CR	NA	5	progression	NA	progression
2	CR	CR	0.048	1	CR	0.048	CR
1	CR	CR	0.022	1	CR	0.005	CR
1	CR	CR	0.073	1	CR	0.074	CR
4	PR	CR	NA	4	PR	0.008	CR
1	CR	CR	0.036	1	CR	0.068	CR
D	-	-	NA	D	-	NA	D
1	CR	CR	0.000	1	CR	0.028	CR
3	CR	CR	0.001	3	CR	0.000	CR
5	PR	PR	5.088	4	PR	10.668	relapse
3	CR	CR	0.025	3	CR	0.041	CR
5	PR	PR	-	3	CR	NA	D (post relapse)
4	PR	CR	0.099	3	CR	0.100	CR
5	PR	PR	NA	5	progression	NA	D
2	CR	CR	0.056	1	CR	0.069	CR
4	PR	CR	NA	4	PR	NA	NA
5	PR	PR	-	5	PR	-	CR
1	CR	CR	0.057	1	CR	0.120	CR
5	PR	CR	0.042	3	CR	0.021	Relapse
5	S	S	NA	5	progression	NA	D
3	CR	CR	0.000	2	CR	0.040	CR
